# The conjugation of SUMO to the transcription factor MYC2 functions in blue light-mediated seedling development in Arabidopsis

**DOI:** 10.1093/plcell/koac142

**Published:** 2022-05-14

**Authors:** Moumita Srivastava, Anjil Kumar Srivastava, Dipan Roy, Mansi Mansi, Catherine Gough, Prakash Kumar Bhagat, Cunjin Zhang, Ari Sadanandom

**Affiliations:** Department of Biosciences, Durham University, Durham DH1 3LE, UK; Department of Biosciences, Durham University, Durham DH1 3LE, UK; School of Life Sciences, University of Dundee, Dundee DD1 5EH, UK; Department of Biosciences, Durham University, Durham DH1 3LE, UK; Department of Biosciences, Durham University, Durham DH1 3LE, UK; Department of Biosciences, Durham University, Durham DH1 3LE, UK; Department of Biosciences, Durham University, Durham DH1 3LE, UK; Department of Biosciences, Durham University, Durham DH1 3LE, UK; Department of Biosciences, Durham University, Durham DH1 3LE, UK

## Abstract

A key function of photoreceptor signaling is the coordinated regulation of a large number of genes to optimize plant growth and development. The basic helix loop helix (bHLH) transcription factor MYC2 is crucial for regulating gene expression in *Arabidopsis thaliana* during development in blue light. Here we demonstrate that blue light induces the SUMOylation of MYC2. Non-SUMOylatable MYC2 is less effective in suppressing blue light-mediated photomorphogenesis than wild-type (WT) MYC2. MYC2 interacts physically with the SUMO proteases SUMO PROTEASE RELATED TO FERTILITY1 (SPF1) and SPF2. Blue light exposure promotes the degradation of SPF1 and SPF2 and enhances the SUMOylation of MYC2. Phenotypic analysis revealed that SPF1/SPF2 function redundantly as positive regulators of blue light-mediated photomorphogenesis. Our data demonstrate that SUMO conjugation does not affect the dimerization of MYC transcription factors but modulates the interaction of MYC2 with its cognate DNA cis-element and with the ubiquitin ligase Plant U-box 10 (PUB10). Finally, we show that non-SUMOylatable MYC2 is less stable and interacts more strongly with PUB10 than the WT. Taken together, we conclude that SUMO functions as a counterpoint to the ubiquitin-mediated degradation of MYC2, thereby enhancing its function in blue light signaling.

## Teaser

SUMO regulates blue light-mediated Arabidopsis seedling development by regulating the DNA binding ability and stability of the bHLH transcription factor MYC2.

## Introduction

Light is the most crucial environmental signal for plant growth and development. Plants deploy two mutually exclusive developmental programs in response to their surrounding light conditions: skotomorphogenesis and photomorphogenesis. In *Arabidopsis thaliana*, the absence of light promotes skotomorphogenesis, which is characterized by elongated hypocotyls, closed cotyledons, and a pronounced apical hook; upon perception of light, hypocotyls shorten and apical hooks straighten, allowing the cotyledons to open. Various photoreceptors are involved in light perception that control these developmental switches, such as phytochromes (phyA–phyE) for far-red and red light, cryptochromes (cry1 and cry2; Ahmad and Cashmore, 1993; Quail, 2002) for blue light, and UVR8 for ultraviolet light (Rizzini et al., 2011). Significant progress has been made in understanding the functions of photoreceptors in mediating these developmental programs in recent years (Ballare et al., 1992; Lin, 2002; Teixeira, 2020).

The coordinated activities of these photoreceptor signaling pathways primarily regulate the expression of a large number of genes involved in light responses, which facilitates the optimization of plant growth and development. Signal transduction after light perception enables a specific set of transcription factors to bring about these gene expression changes. The transcription factor MYC2, which belongs to the bHLH transcription factor family (Z-box binding factors [ZBFs]; Lorenzo et al., 2004), is involved in regulating Arabidopsis seedling development during blue light perception (Yadav et al., 2002; Gangappa et al., 2013; Maurya et al., 2015). Myelocytomatosis 2 (MYC2) is a negative regulator of photomorphogenic growth in blue light (Gangappa and Chattopadhyay, 2010). CONSTITUTIVE PHOTOMORPHOGENIC 1 (COP1) promotes its degradation (Chico et al., 2020) by ubiquitination via PLANT U-BOX PROTEIN 10 (PUB10) (Jung et al., 2015), thereby governing blue light-mediated seedling growth.

Plants have developed efficient, highly sophisticated, and rapid signal transduction mechanisms in response to light that often involve post-translational modifications (PTMs). SUMO modification is a dynamic and reversible PTM that is used to regulate many processes, with central roles in signal transduction mechanisms including protein subcellular localization, protein aggregation, and the control of transcription (Lois et al., 2003; Colby et al., 2006; Catala et al., 2007; Park et al., 2010; Conti et al., 2014). Different classes of small ubiquitin like modifier (SUMO) proteases (Garrido et al., 2018) act by reversing the SUMOylation process, resulting in the removal of SUMO from its target, a process called deSUMOylation. All things being equal, SUMO deconjugation mediated by SUMO proteases affords a mechanism for rapid modulation of molecular pathways that is well suited to light perception and signaling. Indeed, recent studies have shown the significance of a rapidly activated signal in different aspects of plant growth and development, such as hormone signaling and both biotic and abiotic stress responses (Conti et al., 2008, 2014; Mazur and van den Burg, 2012; Bailey et al., 2016; Srivastava et al., 2016a, 2016b, 2017, 2018, 2020, 2021; Hammoudi et al., 2018; Orosa et al., 2018; Orosa-Puente et al., 2018; Rytz et al., 2018). The significance of SUMO in light signal transduction is only just emerging. In particular, we have shown that the SUMO proteases OTS1 and OTS2 govern the balance of conjugation and deconjugation of SUMO to the phyB photoreceptor. SUMOylation inhibits the interaction of the Pfr (active) form of phyB with its immediate signaling partner PHYTOCHROME-INTERACTING FACTOR5. The impaired interaction of these proteins negatively affects photomorphogenic responses; thus, SUMOylation plays a role in desensitizing phyB-mediated signaling (Sadanandom et al., 2015).

Here, we demonstrate that SUMO PROTEASE RELATED TO FERTILITY1 (SPF1) and SPF2, members of the Ubiquitin-like Protease (ULP) family of SUMO proteases, target MYC2 for deSUMOylation during blue light responses in Arabidopsis. We demonstrate that blue light promotes the SUMOylation of MYC2 via the degradation of SPF1/SPF2 SUMO proteases. This contributes to the DNA-binding activity and accumulation of MYC2 by inhibiting its interaction with the ubiquitin E3 ligase PUB10. The subsequent increase in abundance of SUMOylated MYC2 negatively regulates blue light-mediated photomorphogenesis.

## Results

### The Arabidopsis *spf1 and spf2* mutants are affected in blue light-mediated seedling development

The SUMO protease SPF1 was recently shown to be involved in far-red light-mediated regulation of Arabidopsis seedling development. In addition, SPF1 is a positive regulator of blue light-mediated photomorphogenesis (Qu et al., 2020), but the molecular mechanism underpinning the associated hypocotyl elongation in Arabidopsis is still not clear. We used the *spf1 spf2* double mutant to explore the role and significance of SUMO conjugation and deconjugation in Arabidopsis seedling development after blue light perception. To address this issue, we monitored the growth of 6-day-old *spf1 spf2* mutant seedlings in constant darkness, white light, blue light, red light, and far-red light. The *spf1 spf2* mutant showed significantly longer hypocotyls compared to wild-type (WT) in white light as well as blue light irradiation at low fluence but not in red light or far-red light conditions (Figure�1, A–J; Supplemental Figure S1). However, fluence growth curves indicated that *spf1 spf2* seedlings showed elongated hypocotyls in far-red light at higher light intensity (Supplemental Figure S1). These data confirm a previously described role for SPF1 in far-red light-mediated seedling development (Qu et al., 2020). Nevertheless, the molecular mechanism underpinning SPF1 and SPF2 proteins in blue light signaling is not understood. To unravel this SPF-mediated mechanism of the regulation of Arabidopsis seedling development, we analyzed blue light-responsive gene expression by reverse transcriptase-quantitative polymerase chain reaction (RT-qPCR) (Yadav et al., 2002; Gangappa and Chattopadhyay, 2010; Chakraborty et al., 2019) in 6-day-old white light- and blue light-grown seedlings. As shown in Figure�1K and Supplemental Figure S2, the expression of the light-inducible genes was significantly suppressed in *spf1 spf2* mutants compared to WT seedlings specifically in blue light. These data indicate that SPF1/2 SUMO proteases either affect light perception or downstream signaling, including the transcriptional response.

**Figure 1:**
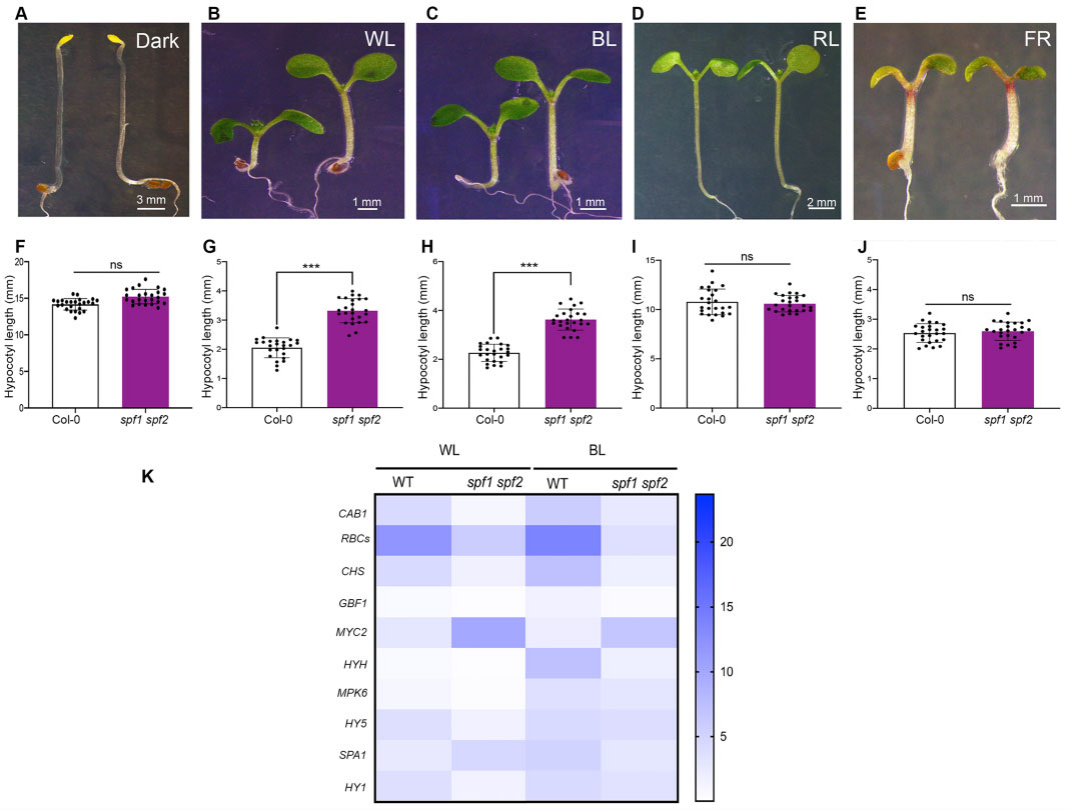
*spf1 spf2* double mutants display hypophotomorphogenic responses specifically to blue light and low-intensity white light. A and F, Representative images and hypocotyl length, respectively, of 6-day-old Col-0 and *spf1 spf2* seedlings grown in the dark. B and G, Representative images and hypocotyl length, respectively, of 6-day-old Col-0 and *spf1 spf2* seedlings grown in constant WL (30 μmol m^−2^ s^–1^). C and H, Representative images and hypocotyl length, respectively, of 6-day-old Col-0 and *spf1 spf2* seedlings grown in constant BL (30 μmol m^−2^ s^−1^). D and I, Representative image and hypocotyl length, respectively, of 6-day-old Col-0 and *spf1 spf2* seedlings grown in constant RL (30 μmol m^−2^ s^−1^). E and J, Representative image and hypocotyl length, respectively, of 6-day-old *Col-0* and *spf1 spf2* seedlings grown in constant FL (5 μmol m^−2^ s^−1^). Values shown are mean � standard deviation (sd) of hypocotyl length in three different biological replicates with 25 seedlings per replicate. *** indicate *P* < 0.0001; significant difference from Col-0. ns, not significant. K, Gene expression heat map of the transcript levels of light-responsive genes measured in Col-0 and *spf1 spf2* mutant seedlings grown in constant WL and BL for 6 days. Intensity of blue color indicates fold changes. RNA was extracted from 6-day-old seedlings grown in the respective light conditions and used for cDNA preparation. RT-qPCR analysis confirmed the hypophotomorphogenic behavior of *spf1 spf2* mutants. BL, blue light; FL, far-red light; RL, red light; WL, white light.

### MYC2 physically interacts with SPF1 and SPF2

Since SUMO proteases such as SPF1 and 2 can target transcriptional regulators and given our finding that *spf1 spf2* double mutants have elongated hypocotyls reminiscent of mutants with suppressed photomorphogenesis, we hypothesized that these SUMO proteases might target transcription factors that influence blue light signaling.

MYC2, a well-established blue light-specific transcription factor, plays a significant role in regulating hypocotyl elongation (Yadav et al., 2002, 2005; Maurya et al., 2015; Chakraborty et al., 2019). This knowledge led us to hypothesize that there might be a physical interaction between SPF1/2 and MYC2. To test this hypothesis, we first conducted bimolecular fluorescence complementation (BiFC) assays (Miller et al., 2015) by co-transfecting different combinations of genes fused to fragments of yellow fluorescent protein (*YFP*) into *Nicotiana benthamiana* leaves to uncover any direct interactions between SPF1/SPF2 and MYC2. Yellow fluorescence signal was detected in the nuclei of *N. benthamiana* cells co-expressing MYC2-YFPn and SPF1/SPF2-YFPc, while the expression of either fusion protein alone did not result in measurable YFP signal (Figure�2A).

**Figure 2:**
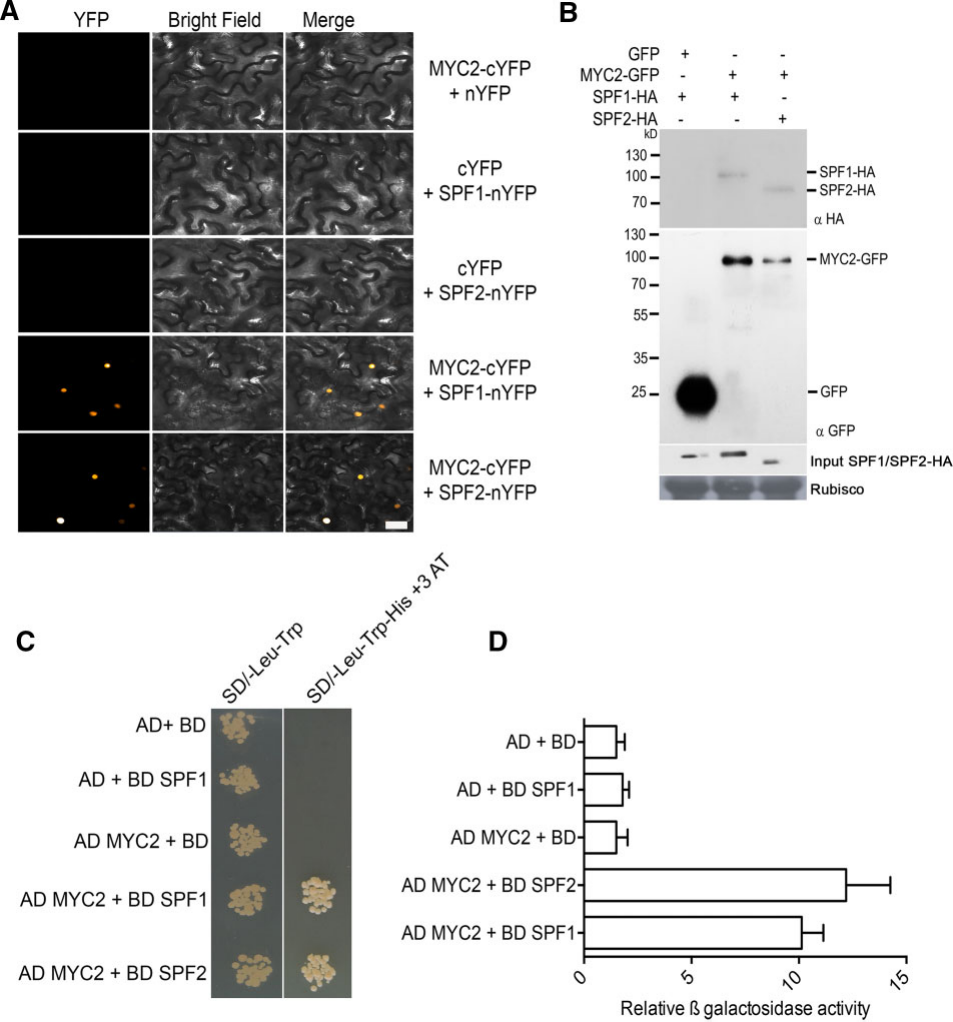
MYC2 physically interacts with SPF1 and SPF2 SUMO proteases. A, Bimolecular fluorescence complementation analysis to show that MYC2 interacts with SPF1 and SPF2 in *N. benthamiana* leaves. Confocal images of epidermal cells of *N. benthamiana* leaves co-expressing the N-terminal domain of enhanced yellow fluorescent protein (SPF1-nYFP or SPF2-nYFP) with the C-terminal domain of YFP (MYC2-cYFP). MYC2-cYFP with nYFP, cYFP with SPF1-nYFP, and cYFP with SPF2-nYFP were used as negative controls. Scale bar: 20 �M. B, MYC2 interacts with SPF1 and SPF2. Proteins from *N. benthamiana* leaves transiently expressing MYC2-GFP with either SPF1-HA or SPF2-HA were collected for immunoprecipitation. Total protein extracts were subjected to immunoprecipitation with anti-GFP antibody beads (IP: αGFP), followed by immunoblot analysis with anti-HA (IB: αHA) antibodies to detect SPF1-HA or SPF2-HA and αGFP (IB: αGFP) antibodies to detect MYC2-GFP. Total proteins from all samples were probed with anti-HA antibody to determine SPF protein levels (SPF1-HA/SPF2-HA). Extract from leaves only expressing GFP was used as a negative control. Ponceau staining for Rubisco was used to ascertain even protein loading in each lane. C, Physical interaction of MYC2 with SPF1/SPF2 by yeast two-hybrid assays. Yeast cells transformed with AD-MYC2 and BD-SPF1 or BD-SPF2 were grown on 2D (-Leu, -Trp) and 4D (-Ade, -His, -Leu, -Trp) selective media. The empty vectors and their combinations with MYC2 and SPF1 or SPF2 were used as negative controls. D, Quantification of the yeast two-hybrid assay results, as shown by relative β-galactosidase activity measurements. The experiment was replicated three times, and data show mean � sd (*n*�=3). BL, blue light.

We next tested the physical interaction between MYC2 and SPF1 or SPF2 by performing transient co-immunoprecipitation (Co-IP) assays. Proteins were extracted from *N. benthamiana* leaves transiently expressing GFP-MYC2 and HA-SPF1 or HA-SPF2 and used for Co-IP analysis. GFP-MYC2 immunoprecipitated HA-SPF1 and HA-SPF2 (Figure�2B). The direct physical interaction between SPF1/2 and MYC2 was confirmed with a yeast two-hybrid assay using their full-length coding sequences as previously described (Srivastava et al., 2015, 2017). A yeast strain containing both *SPF1* and *MYC2* or *SPF2* and *MYC2* was able to grow on synthetic medium lacking His, Leu, and Trp and supplemented with 2-mM 3-AT, in contrast to the negative controls (Figure�2, C and D). The in vivo interaction of SPF1/SPF2 and MYC2 was further supported by confocal microscopy analysis of the nuclear localization of SPF1/SPF2 and MYC2 when SPF1/SPF2-mCherry and MYC2 GFP (Green Fluorescent Protein) were co-expressed in *N. benthamiana* leaves (Supplemental Figure S3).

### Blue light promotes the SUMOylation of MYC2

The altered blue light-dependent photomorphogenic response of the *spf1 spf2* mutants and the strong physical interaction of SPF1 and SPF2 with MYC2 led us to investigate the SUMO status of MYC2 in blue light. Initially, we performed manual in silico analysis of the MYC2 amino acid sequence for peptide motifs containing lysine residues that resemble known SUMO sites in other proteins (Conti et al., 2014, Orosa et al., 2018). Two putative high-confidence SUMO conjugation sites, namely Lysine 109 and Lysine 480, were located in the N-terminal region close to the previously established nuclear localization sequence and the bHLH domain (Supplemental Figure S4A). The potential MYC2 SUMO sites are conserved across plant species from Arabidopsis, rice (*Oryza sativa*), maize (*Zea mays*), potato (*Solanum tuberosum*), and tomato (*Solanum lycopersicum*) to *Brassica rapa* (Supplemental Figure S4B). To ascertain if MYC2 is indeed SUMOylated by blue light treatment in Arabidopsis, we generated transgenic plants harboring 35S promoter-driven GFP-tagged *MYC2* (Supplemental Figure S5) in the *myc2-3* mutant background. The seedlings were grown in the dark and transferred to blue light conditions. SUMO immunoprecipitation assays indicated that blue light treatment rapidly enhanced SUMO conjugation to MYC2 (Figure�3A).

**Figure 3:**
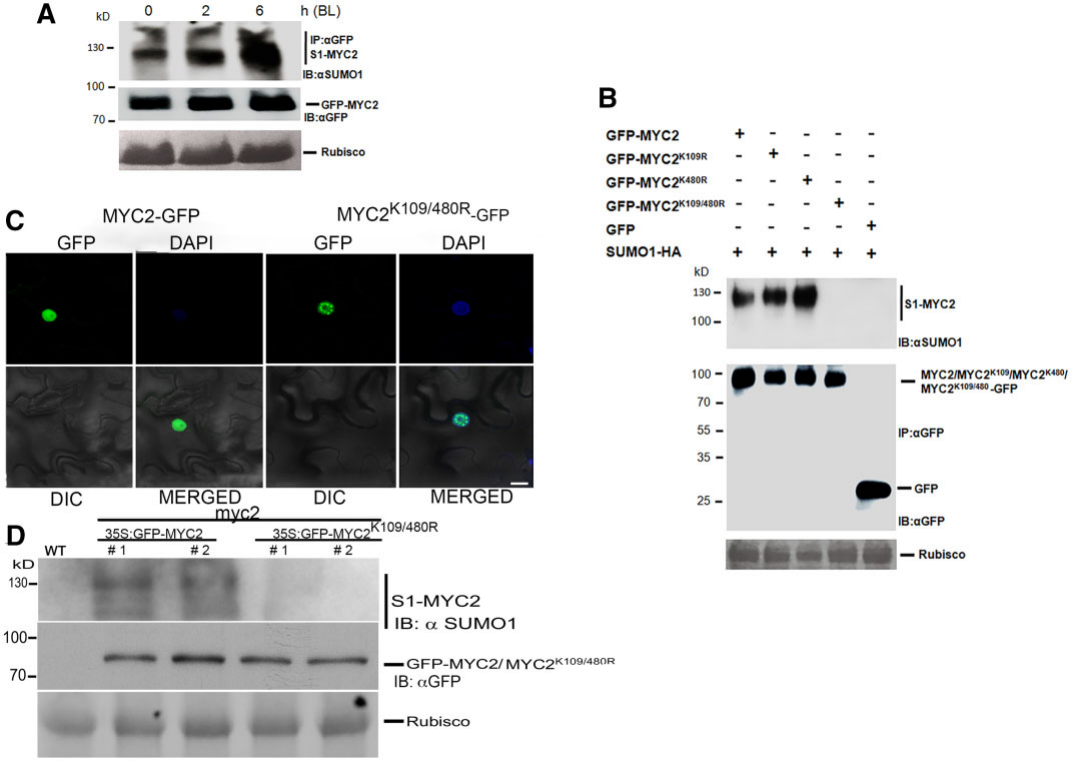
MYC2 is SUMOylated in response to blue light. A, Blue light induces MYC2 SUMOylation. Immunoprecipitation (IP: αGFP) experiments were carried out with αGFP beads from total proteins derived from transgenic lines expressing 35S:MYC2-GFP in the *myc2-3* background after exposure to blue light for 0, 2, and 6 h. Immunoblots were probed with αGFP (IB: αGFP) or αSUMO1/2 (IB: αSUMO1) antibodies. Ponceau staining for Rubisco was used to ascertain even protein loading in each lane. B, MYC2 is SUMOylated at lysine residues 109 and 480 in planta*.* MYC2-GFP or the double lysine mutant fused to GFP (MYC2^2K/R^-GFP) was transiently co-expressed with SUMO1-HA in leaves of *N. benthamiana*. Immunoprecipitation (IP: αGFP) experiments were carried out with αGFP antibody beads from total protein extracts derived from these leaves. Immunoblots were probed with αGFP (IB: αGFP) or αSUMO1/2 (IB: αSUMO1) antibodies. GFP was used as a negative control. Ponceau staining for Rubisco was used to ascertain even protein loading in each lane. C, Confocal imaging of the subcellular localization of MYC2-GFP and MYC22K/R-GFP in *N. benthamiana leaves.* Scale bar, 20�M. D, Lysine residues 109 and 480 in MYC2 are critical for SUMOylation in stable Arabidopsis transgenic lines. Immunoprecipitation (IP: αGFP) experiments were carried out with αGFP antibody beads from total protein extracts derived from transgenic lines expressing *35S:MYC2-GFP* or *35S:MYC2^2K/R^-GFP* in the *myc2-3* background. Immunoblots were probed with αGFP (IB: αGFP) or αSUMO1/2 (IB: αSUMO1) antibodies. Ponceau staining for Rubisco was used to ascertain even protein loading in each lane. BL, blue light.

To ascertain if the in silico identified lysines are real SUMO conjugating sites, we mutated Lysines 109 and 480 in MYC2 to arginines (MYC2^K109/480R^ or MYC2^2K/R^) to maintain the proper amino acid charge but prevent SUMO conjugation (Orosa-Puente et al., 2018; Srivastava et al., 2018, 2020). We next performed Co-IP experiments in *N. benthamiana* via *Agrobacterium*-mediated transient assays with epitope-tagged GFP-MYC2, GFP-MYC2^2K/R^, MYC2^K109^, or MYC2^480R^ single SUMO site mutants and HA-SUMO1. Immunoprecipitation assays indicated that compared to WT MYC2, there was no detectable SUMOylation of MYC2^2K/R^, demonstrating that these conserved lysines are the major sites for SUMO attachment to MYC2 under these conditions (Figure�3B). Co-IP experiments with single SUMO site mutants in MYC2 (MYC2^K109^ or MYC2^480R^) indicated that both lysines were equally important for SUMO conjugation. Therefore, we used MYC2^2K/R^ for further analysis.

To further confirm this finding, we utilized transgenic plants harboring 35S promoter-driven GFP-tagged MYC2^2K/R^ (Supplemental Figure S5), along with the WT version of GFP-tagged MYC2 in the *myc2-3* mutant background to provide more evidence for MYC2 SUMOylation on the identified lysines. Confocal microscopy analysis of the WT MYC2 and SUMO-deficient version of MYC2 (MYC2^2K/R^) did not show any difference in their subcellular localization (Figure�3C). We barely detected any MYC2 SUMOylation in 35S:GFP-MYC2^2K/R^ transgenic plants compared to 35S:GFP-MYC2 with comparable transcript levels (Figure�3D;Supplemental Figure S5). These data indicate that blue light promotes SUMO conjugation onto MYC2 at Lysines 109 and 480.

These results, together with the finding that MYC2 interacts with SUMO proteases SPF1/2, uncover a link between blue light, SUMOylation, and MYC2.

### Blue light promotes the degradation of SPF1 and SPF2

To further understand the role of SPF1 and 2 SUMO proteases in blue light responses, we determined the impact of blue light on SPF1 and 2 protein accumulation. We translationally fused SPF1 and SPF2 with mCherry and MYC2 to GFP. The nuclear localization of mCherry-SPF1 and mCherry-SPF2 was confirmed by confocal microscopy in transient assays in *N. benthamiana* (Figure�4A). mCherry signal was observed by confocal microscopy after exposure to blue light for 4 h. However, SPF1 and 2 fused mCherry signal diminished in the presence of blue light, but the MYC2-GFP signal did not (Figure�4A). We also generated transgenic Arabidopsis plants harboring 35S promoter-driven HA-tagged *SPF1* to monitor protein levels. Protein accumulation of HA-SPF1 was significantly reduced after 4 h of blue light exposure (Figure�4B). To further confirm that the blue light-mediated degradation of SPF1 protein occurred post-transcriptionally, we monitored the transcript levels of the *SPF1* transgene in plants harboring 35S promoter-driven HA-tagged SPF1 (Supplemental Figure S6) and ascertained that they were at similar levels. As expected, immunoblot analysis of *N. benthamiana* transient assays expressing SPF2-HA confirmed that like SPF1- HA, blue light stimulated its degradation (Figure�4C; quantified in Supplemental Figure S7).

**Figure 4:**
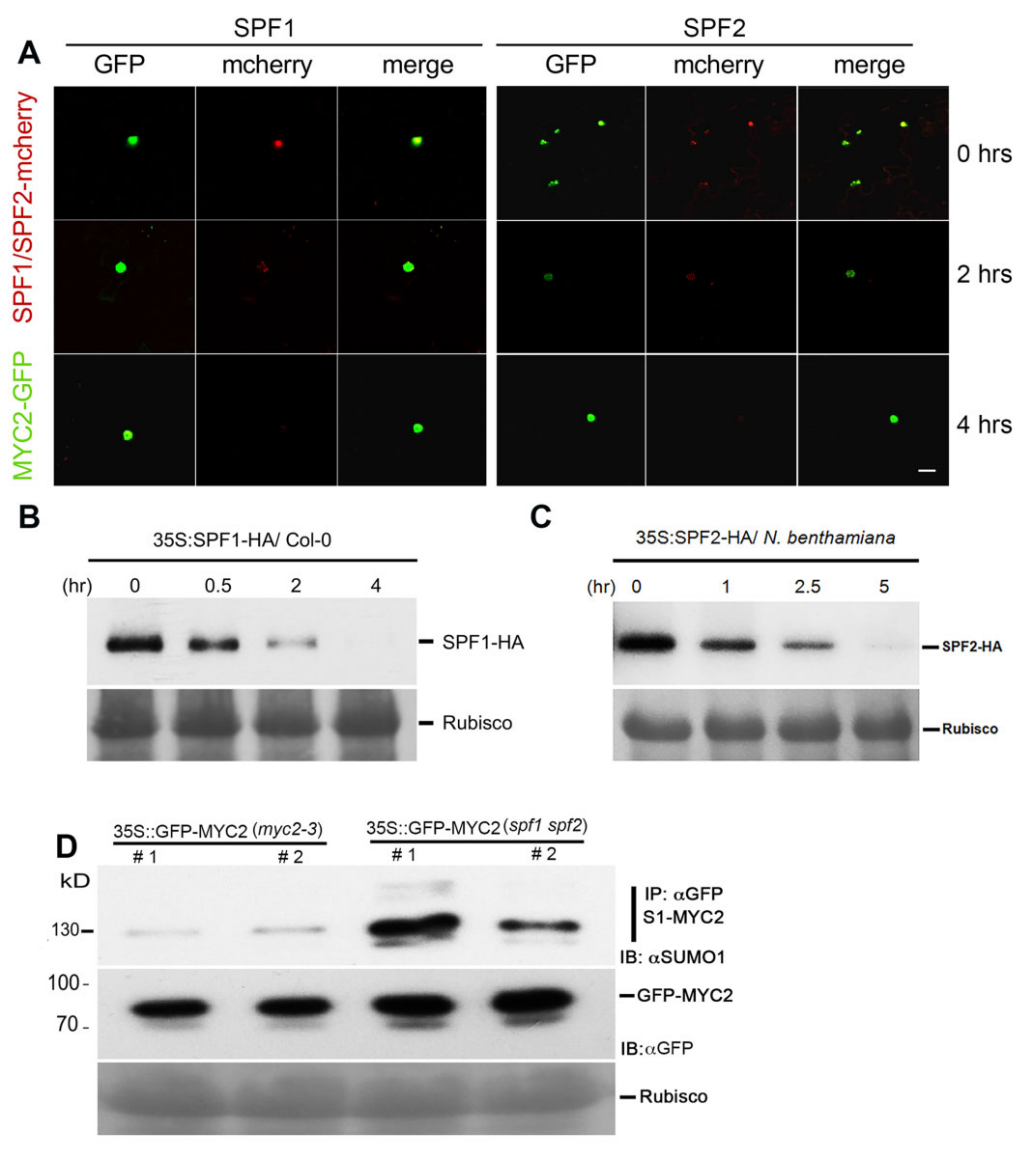
SPF1 and SPF2 colocalize with MYC2 and are destabilized in blue light. A, Confocal imaging reveals that SPF1 and SPF2 colocalize with MYC2 and are destabilized in blue light. *N. benthamiana* leaves co-infiltrated with SPF1-mCherry and MYC2-GFP and SPF2-mCherry and MYC2-GFP, respectively, were analyzed for fluorescence after 3 days. The plants were either used directly for imaging (0 h) or kept in blue light for 4 h before confocal imaging. Images were obtained under a Carl Zeiss Airyscan 880 confocal laser scanning microscope. Scale bar: 20 μm. B, The accumulation of SPF1 is affected by blue light. Ten-day-old 35S:SPF1-HA transgenic Arabidopsis seedlings were exposed to blue light and samples were collected at different time points. Total proteins were extracted from the samples and immunoblotted with anti-HA (IB:αHA) antibody. C, The accumulation of SPF2 is affected by blue light. *Nicotiana benthamiana* leaves transiently expressing 35S:SPF2-HA were exposed to blue light and samples were collected at different time points. Total proteins were extracted from the samples and immunoblotted with anti-HA (IB:αHA) antibody. D, MYC2 is hyperSUMOylated in *spf1 spf2* double mutants. Immunoprecipitation (IP: αGFP) experiments were carried out with αGFP antibody beads from total protein extracts derived from transgenic lines expressing 35S:MYC2-GFP in the *myc2-3* or *spf1 spf12* double-mutant background. Immunoblots were probed with αGFP (IB: αGFP) or αSUMO1/2 (IB: αSUMO1) antibodies. Ponceau staining for Rubisco was used to ascertain even protein loading in each lane.

To further ascertain if SPF1 and 2 affect MYC2 SUMOylation in Arabidopsis, we generated transgenic lines expressing *MYC2-GFP* in the *spf1 spf2* double-mutant background. As expected, we observed enhanced SUMOylation of MYC2- in the *spf1 spf2* double-mutant background compared to *MYC-2-GFP* complementing the *myc2-3* mutant (Figure�4D). Collectively, the evidence indicates that blue light exposure triggers the degradation of the SPF1 and 2 SUMO proteases post-transcriptionally. This finding, together with the discovery that these SUMO proteases interact with MYC2 and that MYC2 SUMOylation showed increased abundance in the *spf1 spf2* double mutant, demonstrates that the enhanced SUMOylation of MYC2 is in large part due to blue light-induced degradation of SPF1/2 SUMO proteases.

### SUMOylation of MYC2 is required for blue light responses in Arabidopsis seedlings

Since MYC2 is a central bHLH transcription factor for blue light-mediated responses (Yadav et al., 2005; Kazan and Manners, 2013; Maurya et al., 2015; Liu et al., 2019), it is plausible that blue light induces the SUMOylation of MYC2 to alter light-dependent gene expression. Indeed, we detected altered blue light-responsive gene expression (Figure�1) in *spf1 spf2* mutants. To further investigate the biological role of blue light-induced MYC2 SUMOylation, we examined the effects of SUMO-deficient mutations on the role of MYC2 in mediating blue light responses in Arabidopsis seedlings. Hypocotyl elongation of transgenic plants expressing SUMO-deficient MYC2 mutant protein was suppressed, which is similar to the response of the *myc2* mutant. In contrast, overexpression of WT *MYC2* was able to restore the *myc2* mutant phenotype (Figure�5, A–D), indicating that SUMOylation is required for MYC2 function in blue light responses. This evidence was substantiated by RT-qPCR data that showed deregulation of blue light-mediated MYC2-dependent gene expression (Figure�5E;Supplemental Figure S8).

**Figure 5:**
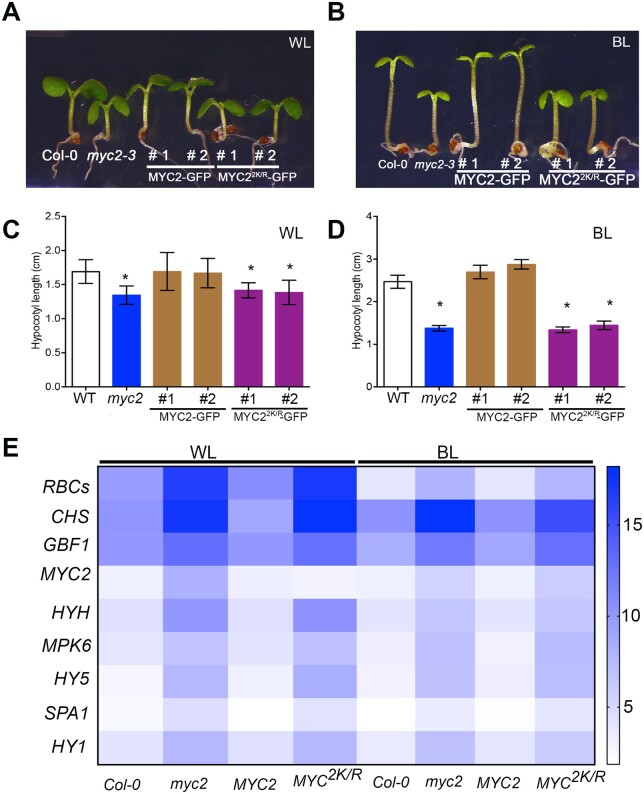
SUMOylation of MYC2 is required to regulate photomorphogenesis. A and C, Representative images and hypocotyl length, respectively, of 6-day-old Col-0, *myc2-3, 35S:MYC2-GFP* and *35S:MYC2^2K/R^-GFP* seedlings grown in constant WL at 30 μmol m^−2^ s^−1^. Error bars indicate standard error (*n* = 20). Asterisks indicate significant difference from Col-0. B and D, Representative images and hypocotyl length, respectively, of 6-day-old Col-0, *myc2-3, 35S:MYC2-GFP* and *35S:MYC2^2K/R^-GFP* seedlings grown in constant BL at 30 μmol m^−2^ s^−1^. Error bars indicate standard error (*n* = 20). Asterisks indicate significant difference from Col-0. E, Gene expression heat map of the transcript levels of light-responsive genes measured in Col-0, *myc2-3, 35S:MYC2-GFP*, and *35S:MYC2^2K/R^-GFP* seedlings grown in constant WL and BL for 6 days. RNA was extracted from 6-day-old seedlings grown in the respective light conditions and used for cDNA preparation. RT-qPCR analysis confirmed that SUMOylation of MYC2 is required to regulate light-responsive gene expression. BL, blue light; WL, white light.

### SUMOylation of MYC2 does not affect its homo/hetero dimerization ability but influences its protein accumulation and ability to bind DNA

The bHLH domain of MYC2 is required to form homodimers with MYC2 and heterodimers with MYC3 (Fernandez-Calvo et al., 2011). One of the SUMO sites we identified was within this bHLH domain (Supplemental Figure S9). Therefore, we hypothesized that SUMO may be involved in the homo/heterodimer formation. To test this hypothesis, we performed transient co-IP analysis in *N. benthamiana* leaves expressing GFP-tagged versions of MYC2 and MYC2^2K/R^ and HA-tagged MYC2 and MYC3. We did not observe any difference in interaction between WT MYC2 and MYC3 with the non-SUMOylatable form of MYC2. We also observed no change in interaction between the single SUMO site mutants harboring WT MYC2. There was also no difference in homodimerization between MYC2^2K/R^. These results indicate that SUMOylation of MYC2 is not involved in the homo/heterodimer formation of these bHLH transcription factors (Figure�6, A–C).

**Figure 6:**
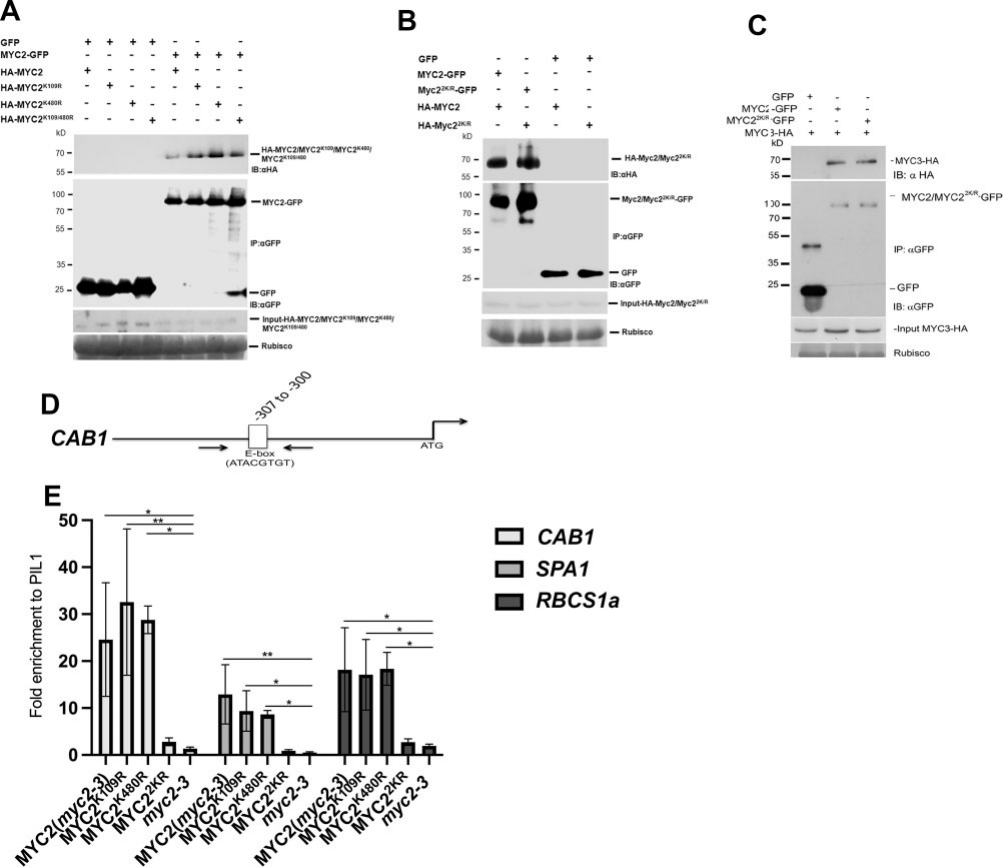
SUMOylation of MYC2 does not affect its homo/hetero dimerization ability but affects its binding to cis-elements. A, SUMOylation of MYC2 is not required for its homodimerization Total proteins were extracted from *N. benthamiana* leaves transiently expressing WT MYC2-HA, single lysine mutants fused to HA (MYC2K109R-HA and MYC2K480R-HA), and double lysine mutant fused to HA (MYC22K/R-HA) with WT MYC2-GFP for immunoprecipitation assays with αGFP antibody beads (IP: αGFP), followed by immunoblot analysis with αHA (IB: αHA) antibodies to detect WT MYC2-HA, MYC2K109R-HA, MYC2K480R-HA, and MYC22K/R-HA along with αGFP (IB: αGFP) antibodies to detect MYC2-GFP. GFP was used as a negative control. Ponceau staining for Rubisco was used to ascertain even protein loading in each lane. B, Total protein from *N. benthamiana* leaves transiently expressing MYC2-GFP with MYC2-HA or MYC22K/R-GFP with MYC22K/R-HA were extracted for immunoprecipitation assays with αGFP antibody beads (IP: αGFP), followed by immunoblot analysis with αHA (IB: αHA) antibodies to detect MYC2-HA/MYC22K/R-HA and αGFP (IB: αGFP) antibodies to detect MYC2-GFP/MYC22K/R-GFP. GFP was used as a negative control. Ponceau staining for Rubisco was used to ascertain even protein loading in each lane. C, SUMO is not required for the heterodimer activity of MYC2. MYC2-GFP or MYC22K/R-GFP was transiently expressed with MYC3-HA in *N. benthamiana*, and coimmunoprecipitation and immunoblotting were performed as described in (A) except that MYC3-HA was analyzed using antibodies against HA epitope. GFP was used as a negative control. Ponceau staining for Rubisco was used to ascertain even protein loading in each lane. D, Schematic representation of the *CAB1* promoter with the E-box marked. E, ChIP assay of MYC2 target gene promoters from *myc2-3*, MYC2-GFP, single SUMO site mutants, and MYC2K/R-GFP transgenic seedlings. ChIP qPCR results are presented as fold enrichment of *SPA1*, *CAB1*, and *RBCS1a* promoter fragments versus that of the negative control binding region, *PIL1*, from MYC2 (*myc2-3*), MYC2K109R, MYC2K480R, MYC22K/R, and *myc2-3* transgenic seedlings. ChIP experiments were repeated three times with similar results. ChIP results of one representative experiment are shown. Error bars represent propagated error value. Asterisks indicate significant difference in multiple comparisons test relative to the control genotype *myc2-3* for each promoter fragment, * *P* ≤ 0.05, **P ≤ 0.01.

To ascertain whether SUMO conjugation influences the DNA-binding activity of MYC2 for light-responsive gene expression, we exploited the promoter elements of MYC2 target genes to perform Chromatin Immunoprecipitation (ChIP) assays with MYC2. MYC2 identifies the E-box element (ATACGTGT) in the promoters of target genes such as *CAB1* (*CHLOROPHYLL A/B-BINDING PROTEIN1*) for binding (Figure�6D) (Gangappa et al., 2013). ChIP assays targeting the E-box within the promoters of the target genes *SUPPRESSOR OF PHYA-105-A 1* (*SPA1*), *CAB1*, and *RBCs* (Rubisco small subunit genes) (Gangappa et al., 2013) with anti-GFP antibodies from transgenic lines harboring WT MYC2, single SUMO site mutants, and MYC2^2KR^–GFP provided quantitative data to analyze the comparative binding of WT and SUMO mutant versions of MYC2 to its target promoters. MYC2^2KR^ consistently showed reduced fold enrichment from target promoters compared to WT MYC2 and the single SUMO site mutants, therefore indicating the involvement of SUMO in better enabling MYC2 to bind to its target gene promoters (Figure�6E). These data provide another line of evidence supporting the importance of SUMOylation for MYC2 function as a transcription factor.

To determine whether this reduced DNA-binding effect observed in the non-SUMO mutants of MYC could compromise its stability, we utilized transgenic lines expressing MYC2–GFP and MYC2^2K/R^–GFP in the *myc2-3* mutant background to ascertain the stability of SUMOylatable and non-SUMOylatable forms of MYC2 proteins. We treated the seedlings with cycloheximide (CHX), an inhibitor of de novo protein synthesis, for up to 2 h and analyzed GFP fluorescence under a confocal microscope. Upon CHX treatment, GFP fluorescence of MYC2^2K/R^–GFP was considerably reduced within an hour compared to WT MYC2 (Figure�7A). To confirm our finding, we performed immunoblot analysis of these lines. Our data indicated that the non-SUMOylatable form of MYC2 was less stable after CHX treatment compared to WT MYC2 protein (Figure�7B). Taken together, our data indicate that SUMOylation of MYC2 is essential for MYC2 stability.

**Figure 7:**
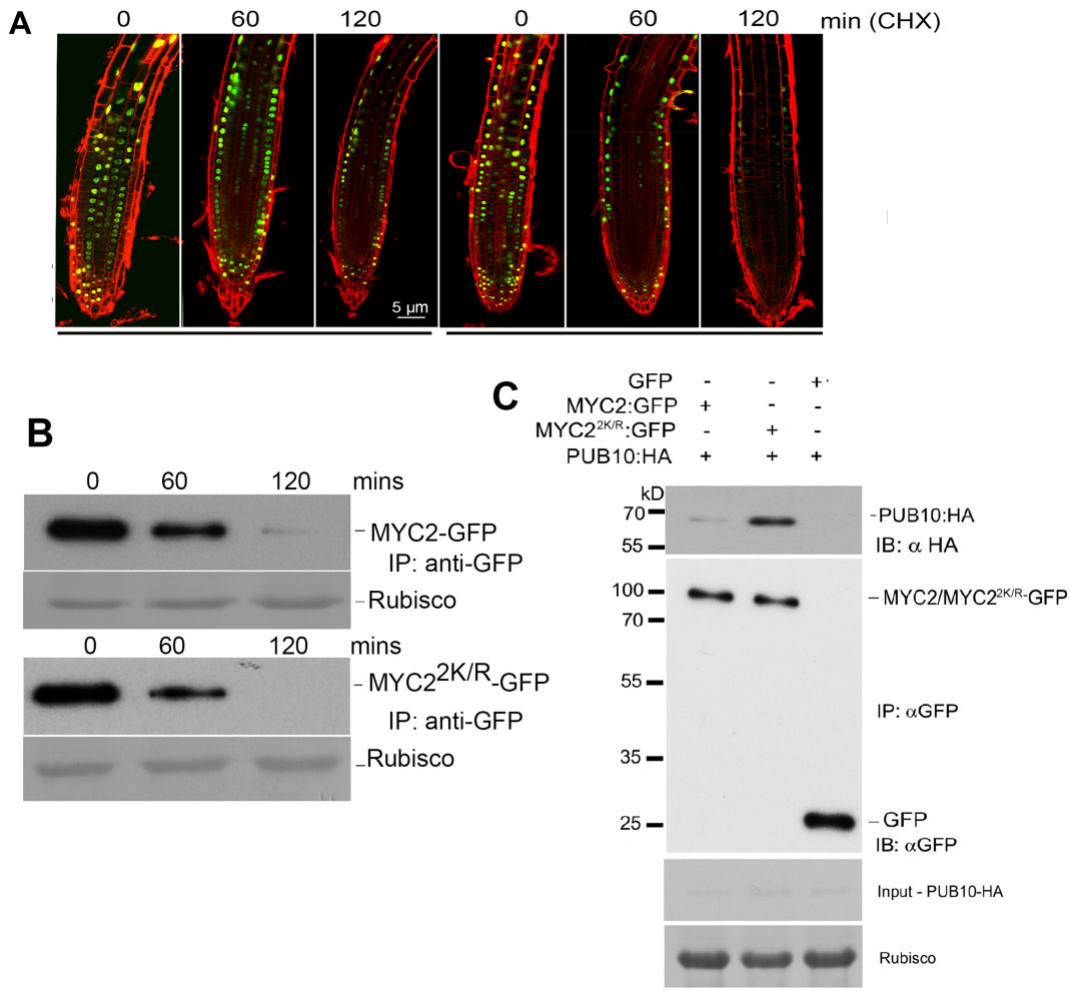
SUMOylation of MYC2 enhances its stability and negatively regulates its interaction with PUB10 E3 ubiquitin ligase. A, Confocal imaging of 7-day-old seedlings of MYC2-GFP and MYC2^2K/R^-GFP treated with 100-�M CHX for 0, 60, and 120 min; GFP fluorescence was monitored after each treatment. Bars = 5�m. B, SUMO contributes to the stability of MYC2. Ten-day-old 35S:MYC2-GFP and 35S:MYC2^2K/R^-GFP seedlings were treated with 100-�M cycloheximide and samples were harvested at 0, 6, and 120 min. Total proteins were extracted from the samples and immunoblotted with αGFP antibody. Ponceau staining for Rubisco was used to ascertain equal protein loading in each lane. C, MYC2-GFP or MYC2^2K/R^-GFP was transiently expressed with AtPUB10-HA in *N. benthamiana*, and coimmunoprecipitation and immunoblotting were performed as described in (A) except that PUB10-HA was analyzed using antibodies against HA epitope. GFP was used as a negative control. Ponceau staining for Rubisco was used to ascertain even protein loading in each lane.

### SUMOylation of MYC2 inhibits its interaction with the E3 ubiquitin ligase PUB10

A recent report demonstrated that MYC2 interacts with the ARM repeat region of the ubiquitin E3 ligase PUB10 for polyubiquitination and degradation (Jung et al., 2015), and SUMOylation is known to regulate ubiquitination (Rott et al., 2017). Therefore, to ascertain whether SUMOylation of MYC2 affects its protein stability, we examined whether the non-SUMOylatable MYC2^2K/R^ was affected in binding to PUB10. Co-IP analysis indicated that the non-SUMOylatable form of MYC2 interacted more strongly with PUB10 compared to WT MYC2 (Figure�7C). This result was further confirmed by comparative analysis of MYC2 and MYC2^2K/R^ ubiquitination. For this experiment, we utilized the GFP-tagged versions of MYC2 and MYC2^2K/R^ transgenic lines. Protein extracts from 10-day-old transgenic seedlings were immunoprecipitated with anti-GFP antibody and examined via immunoblotting using an anti-ubiquitin antibody. The intensity of high molecular size bands corresponding to the ubiquitinated form of MYC2 recognized by the anti-ubiquitin antibody was significantly higher in lines expressing the non-SUMOylatable form of MYC2 (MYC2^2K/R^) than WT MYC2 (Supplemental Figure S10).

## DISCUSSION

Posttranslational modifications are key processes that regulate cellular signaling pathways (Sadanandom et al., 2015; Podolec and Ulm, 2018). SUMO is emerging as a critical PTM in plants with significant roles in plant growth and development in addition to biotic and abiotic stress responses (Miura et al., 2007; Park and Yun, 2013; Srivastava et al., 2016b, 2018, 2021; Orosa et al., 2018). Given the growing list of critical cellular processes affected by SUMO, unlike the ubiquitin system (where hundreds of E3s act as substrate adapters), the SUMO system has only two E3s in Arabidopsis, posing a molecular mystery surrounding the specificity mechanisms of this PTM. Previous studies alluded to the roles played by SUMO deconjugating enzymes (the largest protein family in the SUMO system) in specific adaptive responses in plants. For example, the SUMO protease DeSi3a targets the immune receptor FLS2 to mediate immune responses against bacterial pathogens (Orosa-Puente et al., 2018). ULP1a impinges on the major transcription factor underpinning Brassinosteroid phytohormone signaling to modulate growth under salt stress (Srivastava et al., 2020). SUMO proteases are emerging as key players in light signaling. We previously showed that the double SUMO protease mutant *ots1 ots2* displays hyposensitivity to red light and enhanced phyB SUMOylation, whereas OTS1 de-SUMOylates phyB in vitro and binds to phyB in planta. The data indicated that OTS1 SUMO protease is involved in mediating the reversible SUMOylation of phyB and that the SUMOylation of phyB desensitizes red light-induced signaling (as does phosphorylation of the photoreceptor) (Sadanandom et al., 2015). The SPF1 SUMO protease was recently shown to be an integral part of far-red light signaling pathways by physically interacting with FAR-RED ELONGATED HYPOCOTYL1 (Qu et al., 2020). SPF1 and SPF2 SUMO proteases were reported to be involved in blue light signaling as well, but the molecular mechanism has not been revealed. In the study, we provided evidence for a SUMO-mediated mechanism for blue light signaling in Arabidopsis seedling development via SPF1 and SPF2 SUMO proteases, thereby illustrating specificity in the SUMO system for light signaling via SUMO proteases.

Our phenotypic analysis under various light conditions revealed that SPF1/SPF2 act redundantly as positive regulators of blue light-mediated photomorphogenesis and suppress hypocotyl elongation. Furthermore, we did not observe any difference in hypocotyl elongation under red light (Figure�1, A–C; Supplemental Figure S1). These results strongly suggest a wavelength-specific function of SPF1/SPF2 in the regulation of hypocotyl growth in Arabidopsis seedlings.

The bHLH transcription factor MYC2 plays a critical role as a negative regulator of blue light-mediated photomorphogenesis in Arabidopsis seedlings (Yadav et al., 2005). SPF1/2 physically interacts with MYC2 (Figure�2) and deSUMOylate it. We showed that blue light exposure promotes the degradation of the SUMO proteases SPF1 and SPF2 and that this enhances the SUMOylation of MYC2 (Figures�3, C and 4). Therefore, the increase in MYC2 SUMOylation during blue light exposure (Figure�3C) at least partly functions via the degradation of the SPF SUMO proteases. The mode of enhancing the SUMOylation of different substrate proteins via the degradation of cognate SUMO proteases is an emerging mechanism for substrate specificity in the SUMO modification system (Orosa et al., 2018; Srivastava et al., 2021). Identifying the E3 ubiquitin ligases that mediate the degradation of SUMO proteases could hold the key for improving our understanding of the specificity mechanisms of this PTM.

MYC2 conjugates with SUMO at two different lysines, K139 and K480. Fernandez-Calvo et al. (2011) showed that the bHLH domain of MYC2 is involved in homo- and heterodimerization with MYC3 and MYC4, and our data show that SUMO conjugation does not affect homo/heterodimer formation (Figure�6, A and B). The data suggest that the SUMO sites do not affect the protein interaction surfaces that take part in dimerization. Nevertheless, an intriguing finding is that the non-SUMO form of MYC2 is impaired in binding to the promoters of the light-responsive *CAB* gene. This finding explains the altered blue light-responsive gene expression profiles of transgenic lines expressing MYC2^2KR^ compared to WT MYC. However, the *MYC2* transgene in these complemented lines in the *myc2-3* background was driven by the 35S promoter, which might affect target gene expression. One key reason for using the 35S promoter was to understand the role of PTMs of MYC2. Lines harboring transgenes driven by the 35S promoter lines are ideal for this type of analysis, as they remove potential complications from genes driven by their own promoters, which might produce altered gene expression when responding to various environmental cues, especially for transcription factors that modulate their own expression. Hence, we used the constitutive 35S promoter to rule out changes in *MYC2* at the transcriptional level.

Furthermore, non-SUMO MYC2 showed enhanced interactions with the E3 ubiquitin ligase PUB10 (Figure�6C), which was previously shown to target MYC2 for ubiquitination and degradation (Jung et al., 2015). Therefore, it is likely that SUMO conjugation to MYC2 enables the transcription factor to maintain DNA binding and therefore becomes less of a target for PUB10 ubiquitination. Our data reveal a facet to MYC2 protein regulation via SUMO that connects DNA binding to ubiquitination. Taken together, we showed that blue light exposure promotes the SUMOylation of MYC2 to inhibit PUB10-mediated ubiquitination of MYC2 and ultimately MYC2 accumulation (Figure�8), leading to the regulation of blue light-mediated gene expression.

**Figure 8:**
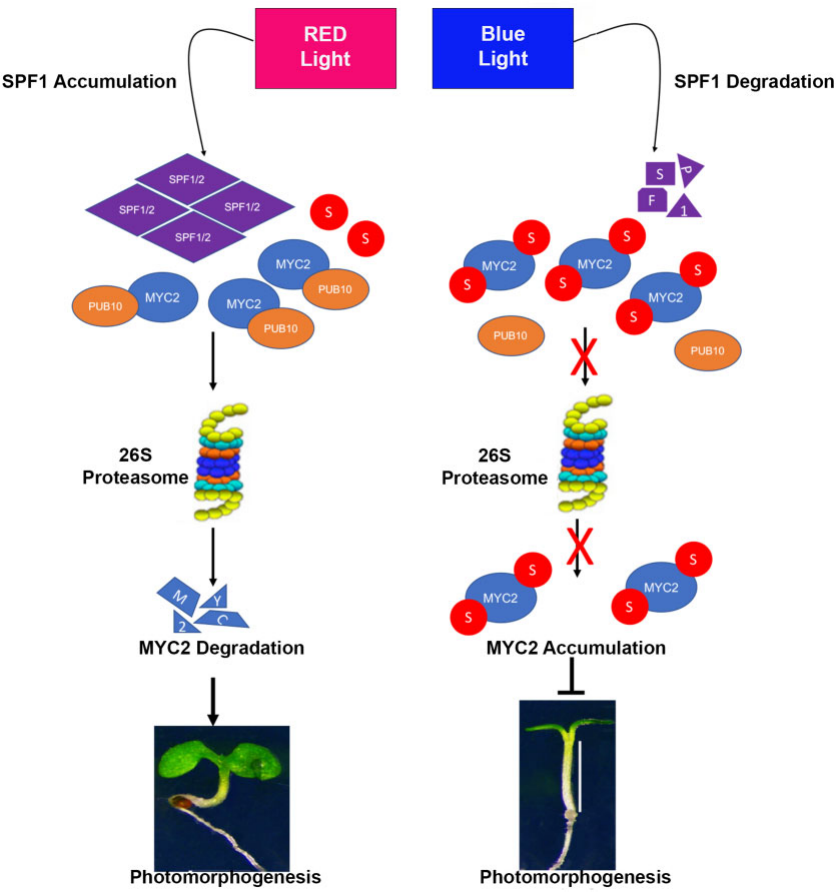
A schematic diagram to illustrate the role of SUMO in regulating photomorphogenesis through MYC2. In red light, SUMO proteases SPF1 and SPF2 accumulate, causing deSUMOylation of MYC2. The deSUMOylated MYC2 is targeted for the proteasomal degradation pathway, resulting in hyperphotomorphogenic responses in Arabidopsis seedlings. In blue light, SPF SUMO proteases are degraded, causing SUMOylation of MYC2, which promotes its accumulation and enhanced DNA binding, resulting in elongated hypocotyls in Arabidopsis seedlings.

The SUMO targeted lysines that we identified in MYC2 are conserved in two other closely related bHLH transcription factors, MYC3 and MYC4 (Supplemental Figure S4A). These sites are also conserved across plant species, as shown in Supplemental Figure S3. From an evolutionary point of view, it will be interesting to understand the functional significance of MYC2 SUMOylation across plant species, especially in crop plants.

Our data identify MYC2 as a bonafide SPF1/2 substrate for deSUMOylation, thereby forming a direct mechanistic link between SUMO and blue light-mediated regulation of gene expression to integrate seedling growth with its environment. Aside from light signaling, MYC2 functions as a point of crosstalk in multiple signaling pathways, including the abscisic acid, jasmonic acid, and ethylene pathways (Abe et al., 2003; Anderson et al., 2004; Sethi et al., 2014). As such, it will be intriguing to ascertain whether SUMOylation of MYC2 is critical in this crosstalk.

## Materials and methods

### Plant materials and growth conditions

*Arabidopsis* *thaliana* plants (Columbia ecotype genetic background) were used as the WT in all experiments. All Arabidopsis plants in soil (John Innes No. 2 compost; medium clay content, contains peat, perlite, 1% nutrient salts, trace elements and iron, pH 5.8) were grown in a climate chamber (Panasonic) in 60% relative humidity, with a constant temperature of 22�C and under a 16-h/8-h day/night cycle with a daytime light intensity of 120–150 �mol photons m^–^^2^ s ^–^^1^ (the climate chamber was equipped with LuxLine Plus F36W 830 Warm White de Luxe fluorescent tubes). T-DNA mutants for *spf1* and *spf2* were obtained from the Eurasian Arabidopsis Stock Centre, and homozygous lines were selected by genotyping. Seeds were surface sterilized, stratified at 4�C for 3 days, and germinated on 1/2 mass spectrometry (MS) plates and 0.8% agar, with or without treatments as indicated. All Arabidopsis on plates were grown in climate chambers as indicated above.

### Image quantification

For morphological studies, plants were placed onto 0.8% agar plates and photographed. Hypocotyl length measurements were then made using Image J. Immunoblot band intensities were quantified by measuring pixel intensity in Image J.

### Plasmid construction

All constructs were generated by GATEWAY cloning technology. To generate the GFP-MYC2, HA-SPF1, HA-SPF2, SPF1-mCherry, and SPF2-mCherry constructs, the corresponding cDNA fragments were PCR amplified and cloned into pENTRD-TOPO vector. By homologous recombination, all genes were transferred into their final vector pEG104 (GFP), pEG201 (HA), pMDC43 (mCherry). All constructs were confirmed by sequencing (Supplemental Table S1).

### Site-directed mutagenesis

Mutated versions of *MYC2* were generated by site-directed mutagenesis using the pENTR/D-TOPO clones as template. Oligonucleotide primers used to introduce the mutations are listed in Supplemental Table S1. The introduction of mutations was confirmed by sequencing, performed both before and after introduction of the mutated *MYC2* coding sequences into pEarleyGate destination vectors using LR Clonase (Invitrogen).

### Generation of transgenic plants

To generate transgenic plants, the 35S:GFP-MYC2 or 35S:GFP-MYC2^2K/R^ constructs were introduced into *Agrobacterium tumefaciens* strain GV3101:pMP90, which were used to transform Arabidopsis *myc2-3* plants via the floral dip method as previously described (Conti et al., 2008). The transgenic plants were selected using BASTA and progressed to the T3 generation before they were used for analysis.

### RNA extraction, cDNA synthesis, and RT-qPCR

Total RNA extraction was carried out from 10-day-old seedlings grown on 1/2 MS plates using a Sigma Aldrich RNA isolation kit (Sigma-Aldrich) following the manufacturer’s protocol. RNA purity and quantity were measured with a NanoDrop Spectrophotometer (NanoDrop 2000c, Thermo Fisher Scientific, USA). One microgram of RNA was treated with RQ1 RNase-free DNase (Promega) and subjected to retro transcription with superscript III reverse transcriptase (Thermo Fisher Scientific) and oligo-dT, according to manufacturer’s protocol. RT-qPCR analysis was performed using the Light Cycler 480 SYBR Green Master Mix (Roche, Switzerland) and carried out using Qiagen Rotor-Gene Q (USA) following the manufacturer’s procedures. Gene-specific nucleotides used to perform RT-qPCR are listed in Supplemental Table S1. RT-qPCR was performed on at least three experimental replicates per time point using *ACTIN* as a reference gene.

#### Immunoprecipitation, Co-IP, and immunoblotting

Total protein was extracted from the samples for immunoprecipitation using extraction buffer containing 100-mM Tris–HCl, pH 8.0, 0.1% [w/v] sodium dodecyl-sulfate (SDS), 0.5% [w/v] sodium deoxycholate, 1% [v/v] glycerol, 50-mM sodium metabisulphite, 100-mM dithiothritol, and protease inhibitor cocktail (Roche, Switzerland). Immunoprecipitation with anti-GFP was performed. Total protein was incubated with 50-�l anti-GFP beads (Chromotek anti-GFP beads) and incubated on ice for 30 min. The beads were centrifuged at 10,000*g* for 1 min and washed three times with 1 mL of cold IP buffer. After the last wash, 50 μL of pre-heated (95�C) 1 � SDS-loading buffer was used to elute the immuno-complex, which was analyzed by 10% sodium dodecyl-sulfate polyacrylamide gel electrophoresis (SDS–PAGE) using immunoblotting methods with rabbit polyclonal anti-GFP antibody (Abcam, Cat. No. ab6556, dilution: 1:6,000), anti HA antibody (Roche, dilution 1:6,000), anti-Ub (Sigma 1:1,000), and anti-SUMO1/2 antibodies generated against *At*SUMO1 in rabbit. The experiments were repeated at least three times.

The protein concentration was determined using a Direct Detect Infra-red Spectrometer (EMD Millipore), and sample concentrations were equalized by adding extraction buffer. Protein loading dye (4�) was added, and the samples were separated on polyacrylamide gels. The proteins were transferred to a polyvinylidene difluoride membrane, blocked with 5% semi-skimmed milk powder at room temperature, and probed with the respective antibodies. Secondary horseradish peroxidase-conjugated antibodies were applied before imaging the blots with X-ray film using an automated developer.

### Yeast two-hybrid assay

Yeast two-hybrid assays were performed as described in Srivastava et al. (2016b) using the appropriate plasmids that contained the indicated genes of interest. To investigate protein–protein interactions, *SPF1* and *SPF2* were individually cloned into pDEST22 to produce translational fusion proteins with the GAL4 DNA activation domain. However, full-length *MYC2* was cloned into pDEST32 to produce translational fusion proteins with the GAL4 DNA-binding domain. Yeast strain AH109 was used to test for interactions on triple-dropout medium lacking Trp, Leu, and His with 3-AT (Srivastava et al., 2016b). The protein–protein interactions were also examined by β-galactosidase assays using chlorophenol red-β-d-galactopyranoside as a substrate. The relative β-galactosidase activities were calculated according to the manufacturer’s instructions (Clontech Laboratories).

### Confocal microscopy and colocalization assay

Confocal microscopy was performed using a Zeiss 880 Airyscan system. At least five roots for transgenic Arabidopsis lines were analyzed for each treatment in each experiment in three independent biological replicates (separate experiments). Four-week-old *N.* *benthamiana* plants were infiltrated on the abaxial side of the leaf with infiltration medium (10-mM MgCl_2_ and 150-mg ml^–1^ acetosyringone) or *A.* *tumefaciens* suspended in infiltration medium. *Agrobacterium* cultures were prepared following a standard protocol. *Agrobacterium* harboring expression constructs were infiltrated at an optical density (OD)_600_ of 0.1 into *N. benthamiana* 72 h prior to confocal imaging. Sections of *N. benthamiana* leaves transiently expressing MYC2-GFP or GFP only and/or SPF1/SPF2-mCherry proteins were randomly sampled and mounted in water. Imaging was conducted with a Zeiss LSM 880 laser scanning confocal microscope with an Airyscan module. The excitation wavelength was 488 nm for GFP and 594 nm for mCherry. Emission was detected using BP 495–550 nm for GFP and an LP 605-nm filter for mCherry. Airyscan processing was done using automatic Weiner filter settings.

### BiFC assay

*Nicotiana* *benthamiana* leaves were co-infiltrated with *A.* *tumefaciens* GV3101 cells containing the indicated plasmids or combination of plasmids for the BiFC assays. The BiFC assays were performed as described in Hu et al. (2002). We used the vectors pYFN43 and pYFC43 to clone *SPF1/2* and *MYC2*, respectively (Belda-Palaz�n, 2020). Epidermal cells of transformed leaves of at least three or four plants were used for the assays. A minimum of three repeats was performed for each construct.

### Chromatin immunoprecipitation assay

ChIP was conducted as described in the Chromotek protocol for *A. thaliana* with the following modifications: 1 g (fresh weight) of 12-day-old *myc2-3*, 35S:GFP-MYC2, and 35S:GFP-MYC2^2K/R^ Arabidopsis seedling tissues were harvested and cross-linked for 15 min under a vacuum in 1% formaldehyde on ice. For processing, samples were sonicated using a Covaris M220 ultrasonicator (peak power = 75.0, duty factor = 5.0, cycles/burst = 200, at 7�C) for 6 min. Five percent volume was taken as input, and the remaining sample volume was incubated with 25-μL anti-GFP beads (Chromotek) for 2 h at 4�C. The samples were washed and sample elution was carried out according to the manufacturer’s protocol. One microliter of input or immunoprecipitated sample was used for qPCR as mentioned above. Primer pairs used were: SPA1 FP ChIP, GGCTGAGCCCAAAAAGTGTT; SPA1 RP ChIP, AGTGTTGCATGGGGGAAGTA; RBCS1α FP ChIP, AAAGGGCCAAGTCCACCAG; RBCS1α RP ChIP, CCACAAAGGCCTAAGGAGAG; PIL1 FP ChIP, AGCAGAGAAAAATGGAGCGGT; PIL1 RP ChIP, GATAAAGAGTAGAAGTATTC; CAB1 FP ChIP, GATGCTCTCAGGATTTCATA; CAB1 RP ChIP, CTTCGCAGATTCGCAATTGA.

### Quantification and statistical analysis

Statistical analysis was performed using GraphPad Prism8 (GraphPad Software). The values shown in the figures are either means of three experimental replicates or means of three independent experiments as specified in each figure caption. Student’s *t* test, one-way and two-way analysis of variance (ANOVAs) were performed in Graph-pad Prism (Supplemental File S1). Details of statistical tests are indicated in each figure legends.

### Accession numbers

Sequence data from this article can be found in the GenBank/EMBL libraries under the following accession numbers: SPF1; AT1G09730, SPF2; AT4G33620, MYC2; AT1G32640.

## Supplemental data

The following materials are available in the online version of this article.

**Supplemental Figure S1.** *spf1 spf2* mutant shows hypophotomorphogenic responses under white light, blue light, and far-red light.

**Supplemental Figure S2.** Expression analysis of *SPF1* and *SPF2* in *Col-0*.

**Supplemental Figure S3.** SPF1 and SPF2 co-localize with MYC2 in *N. benthamiana* transient assays.

**Supplemental Figure S4.** SUMO sites of MYC2 are conserved in different plant species.

**Supplemental Figure S5.** Expression analysis of *MYC2* in 35S: MYC2-GFP (*myc2-3*) transgenic lines.

**Supplemental Figure S6.** Expression analysis of *SPF1* in 35S: SPF1-HA (*Col-0*) transgenic lines.

**Supplemental Figure S7.** Quantification of SPF1-HA and SPF2-HA in the immunoblots in Figure�4, B and C using ImageJ software.

**Supplemental Figure S8.** Expression analysis of light-responsive genes.

**Supplemental Figure S9.** Schematic diagram depicting the organization of the MYC2 structural domains.

**Supplemental Figure S10.** Immunoblot detecting ubiquitination to illustrate the comparative ubiquitination of MYC2-GFP and MYC2^2K/R^-GFP in stable Arabidopsis transgenic lines.

**Supplemental Table S1.** List of DNA oligonucleotides used in the study.

**Supplemental File S1.** ANOVA tables.

## Supplementary Material

koac142_Supplementary_Data

## Notes

M.S., A.K.S., D.R., M.M., P.K.B., C.G., and A.S. designed the research and analyzed the data. M.S., A.K.S., D.R., M.M., P.K.B., C.G., C.Z. performed most of the experiments. M.S., A.K.S., D.R., M.M., P.K.B., and A.S. wrote the paper. All authors read and commented on the manuscript.

The authors responsible for distribution of materials integral to the findings presented in this article in accordance with the policy described in the Instructions for Authors (https://academic.oup.com/plcell) are: Anjil Kumar Srivastava (asrivastava001@dundee.ac.uk) and Ari Sadanandom (ari.sadanandom@durham.ac.uk).
