# Supplementary material for: The conjugation of SUMO to the transcription factor MYC2 functions in blue light-mediated seedling development in Arabidopsis
Source: Plant Cell. 2022 May 14;34(8):2892–906. doi: 10.1093/plcell/koac142 (PMC9338799; doi:10.1093/plcell/koac142)
Supplement: koac142_Supplementary_Data [file koac142_supplementary_data.zip › koac142-suppl_data/tpc.21.00535_SupplementalFiguresandTables.pdf]

Supplemental Data. Srivastava et al. (2022). The conjugation of SUMO to the transcription factor MYC2 functions in blue light-mediated seedling development in Arabidopsis. Plant Cell.

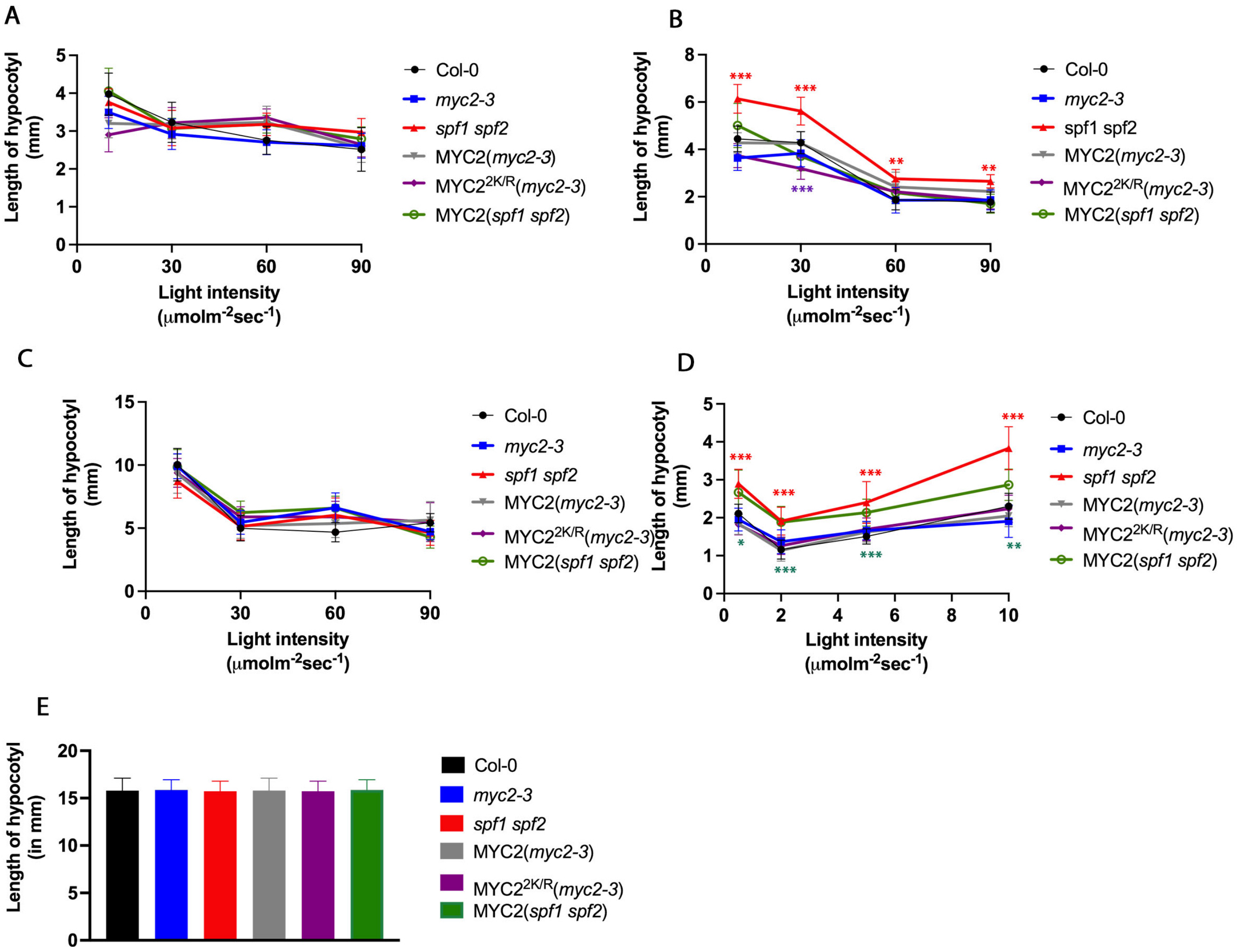

Supplemental Figure S1. The *spf1 spf2* mutant shows hypophotomorphogenic responses under white light, blue light, and far-red light (supports Figure 1). (A-D) Fluence curves of hypocotyl lengths of six-day-old *Col-0*, *myc2-3, spf1 spf2*, MYC2 (*myc2-3*), MYC2KR (*myc2-3*), MYC2 (*spf1 spf2*) grown in different fluence intensities of constant white light, blue light, red light (10, 30, 60 and 90 µmolm-2sec-1), far-red light (0.5, 2, 5, and 10 µmolm-2sec-1) and (E) in the dark. Asterisk*, **, *** indicate P<0.05, <0.005, and <0.0005, respectively.. Error bars represent standard deviation and N=10(N stands for number of replicates).

Supplemental Data. Srivastava et al. (2022). The conjugation of SUMO to the transcription factor MYC2 functions in blue light-mediated seedling development in Arabidopsis. Plant Cell.

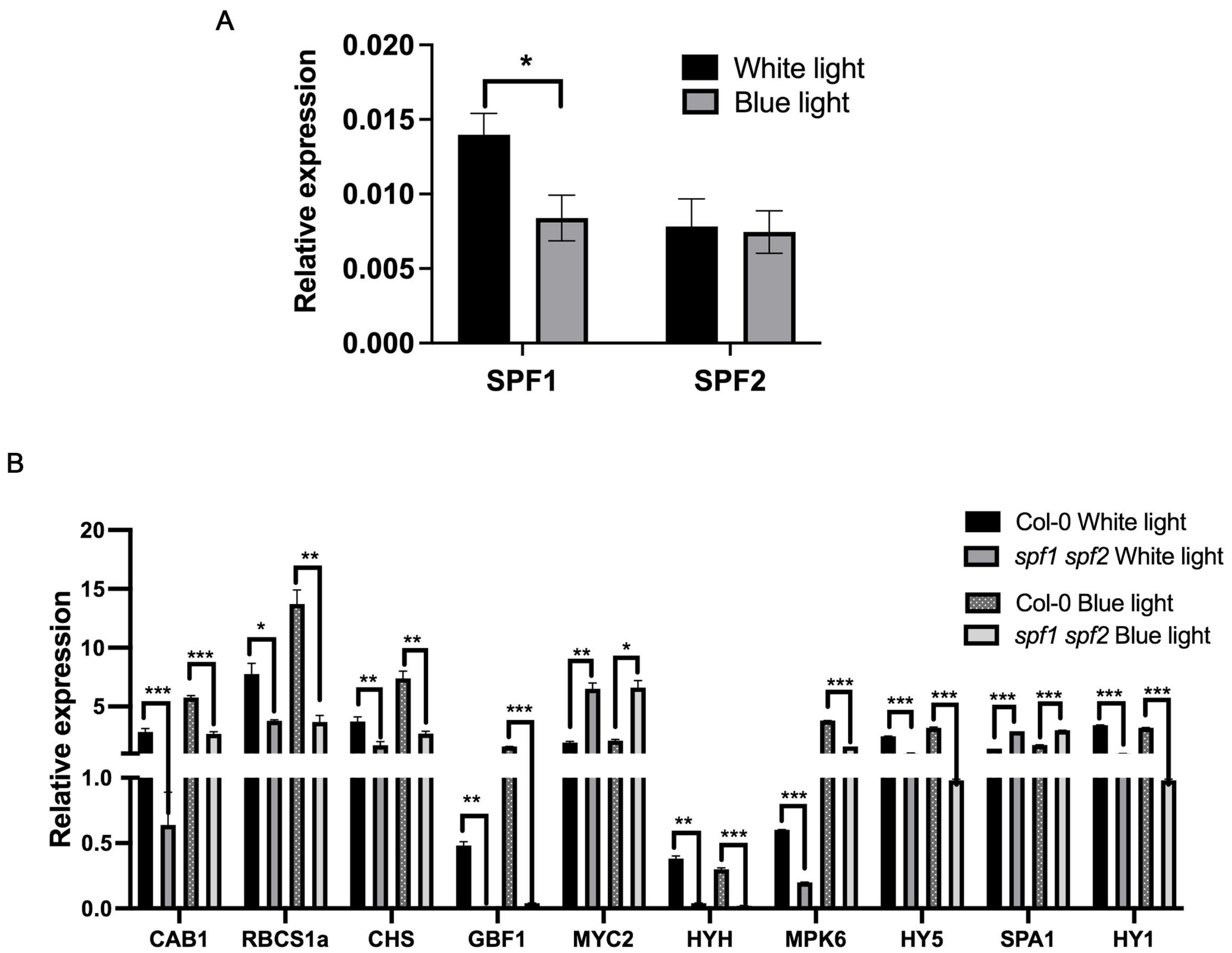

Supplemental Figure S2. Expression analysis of *SPF1* and *SPF2* in Col-0(supports Figure 1). (A) Expression levels of *SPF1* and *SPF2* were compared in Col-0 under white light and blue light treatment via RT-qPCR ($P<0.05$, Student's t-test). (B) Expression levels of light-responsive genes measured in Col-0 and *spf1 spf2* mutant seedlings grown in constant white light or blue light for six days. Asterisk*, **, *** indicate P<0.05, <0.005, and <0.0005, respectively. Error bars represent standard deviation and N=3(N stands for number of replicates).

Supplemental Data. Srivastava et al. (2022). The conjugation of SUMO to the transcription factor MYC2 functions in blue light-mediated seedling development in Arabidopsis. Plant Cell.

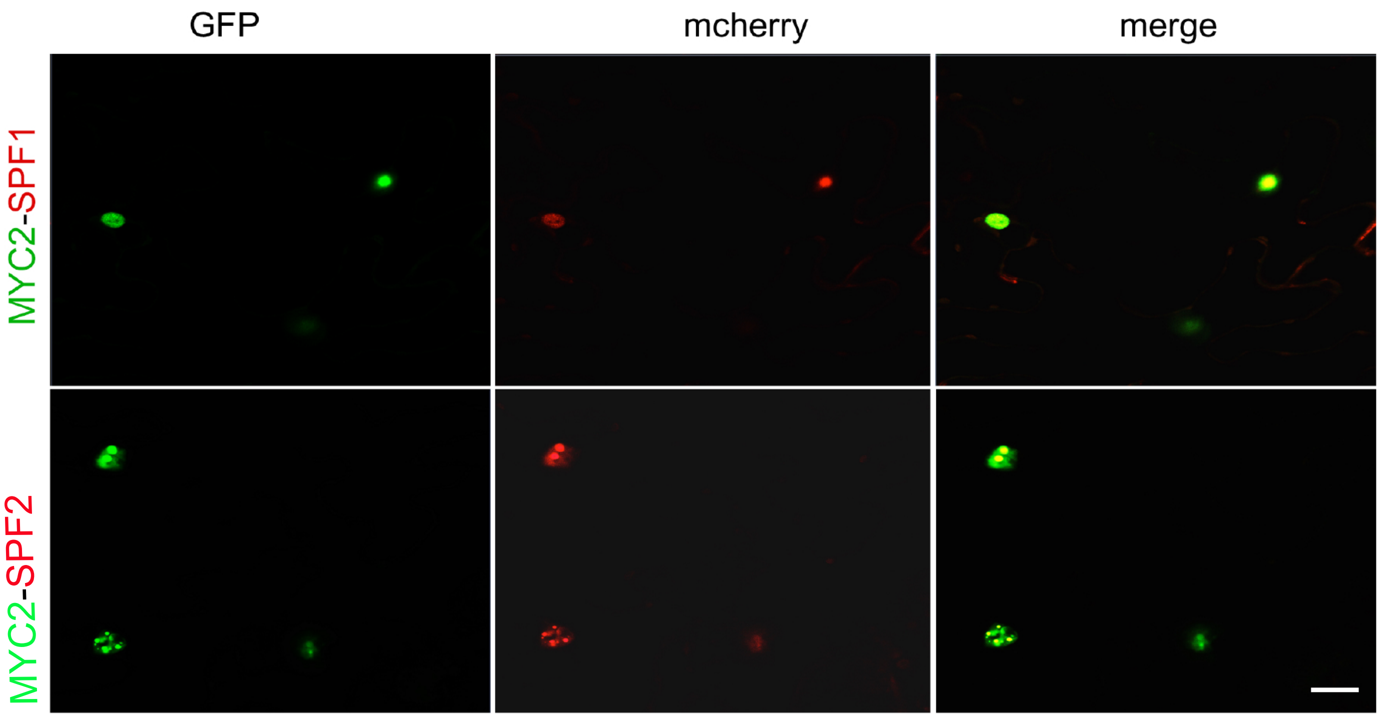

Supplemental Figure S3. SPF1 and SPF2 co-localize with MYC2 in N. benthamiana transient assays (supports Figure 3).

N. benthamiana leaves co-infiltrated with SPF1-mCherry and MYC2-GFP and SPF2-mCherry and MYC2-GFP, respectively, were analyzed for fluorescence after 3 days by confocal microscopy. Images were obtained under a Carl Zeiss Airyscan 880 confocal laser scanning microscope. Scale bar, 20 µm.

A

```
MYC2_A    LGWGDGYYKGEEDKANPRRRSSSPPFSTPADQEYRKKVLRELNSLISGGVAPSDDAVDEE    160
MYC3_A    LGWGDGYYKGEEDKEKKK--N----NTNTAEQEHRKRVIRELNSLISGGIGVSDESNDEE    143
MYC4_A    LGWGDGYYKGEEEKSRKKKSN----PASAAEQEHRKRVIRELNSLISGGVGGGDEAGDEE    158

MYC2_A    GRKPANGREEPLNHVEAERQRREKLNQRFYALRAVVPNVSKMDKASLLGDAIAYINELKS    499
MYC3_A    GRKPANGREEPLNHVEAERQRREKLNQRFYSLRAVVPNVSKMDKASLLGDAISYINELKS    462
MYC4_A    GRKPANGREEPLNHVEAERQRREKLNQRFYSLRAVVPNVSKMDKASLLGDAISYISELKS    463
```

B

```
OsMYC2    TGASLLGWGDGYYKGCDDDKRK-QRSSTPA--AAAEQEHRKRVLRELNSLIAGAGAAPDE    205
ZmMYC2    TGASLLGWGDGYYKGCDEDKRK-QKPLTPS--AQAEQEHRKRVLRELNSLISGAAAAPDE    145
SlMYC2    SSPSVLGWGDGYYKGEEDKAKRKLSVSSPAYI--AEQEHRKKVLRELNSLISGAPPGTDD    180
StMYC2    SSPSVLGWGDGYYKGEEDKAKRKLAVSSPAYI--AEQEHRKKVLRELNSLISGAPAGTDD    180
AtMYC2    SGASVLGWGDGYYKGEEDKANPRRRSSSPPFSTPADQEYRKKVLRELNSLISGGVAPSDD    155
BrMYC2    SGASVLGWGDGYYKGEEDKAKPRQRTSPPPFSTPADQEYRKKVLRELNSLISGGCGPTDD    143

OsMYC2    VVPNVSKMDKASLLGDAISYINELRGKLTALETDKETLQSQMESLKKERDARPPAPSG--    655
ZmMYC2    VVPNVSKMDKASLLGDAISYINELRGKLTSLETDKETLQTQVEALKKERDARPPSHSAGL    608
SlMYC2    VVPNVSKMDKASLLGDAISYINELKSKLQNTESDKEDLKSQIEDLKKESRRPGPPPPPNQ    594
StMYC2    VVPNVSKMDKASLLGDAISYINELKSKLQNTESDKEDLKSQIEDLKKESRRPGPP-PPNQ    595
AtMYC2    VVPNVSKMDKASLLGDAIAYINELKSKVVKTESEKLQIKNQLEEVKLELAGRKASASGGD    533
BrMYC2    VVPNVSKMDKASLLGDAIAYINELKSKVTKTESEKTQIKTQLEEVKMELAGRKASA-GGD    513
```

Supplemental Figure S4. SUMO sites of MYC2 are conserved in different plant species (supports Figure 3).
(A) SUMO sites are conserved in MYC transcription factors (MYC2, MYC3, and MYC4). Amino acid alignment of MYC2, MYC3, and MYC4 in Arabidopsis thaliana (At). The conserved lysines at the two positions are highlighted in gray.
(B) Amino acid alignment of MYC2 from rice (Oryza sativa; OsMYC2), maize (Zea mays; ZmMYC2), tomato (Solanum lycopersicum; SlMYC2), potato (Solanum tuberosum; StMYC2), Arabidopsis thaliana (AtMYC2), and Brassica rapa (BrMYC2). The conserved lysines at the two positions are highlighted in gray. The Phytozome (http://phytozome.jgi.doe.gov/pz/portal.html) database was used to obtain the MYC2 homolog sequences from different plant species.

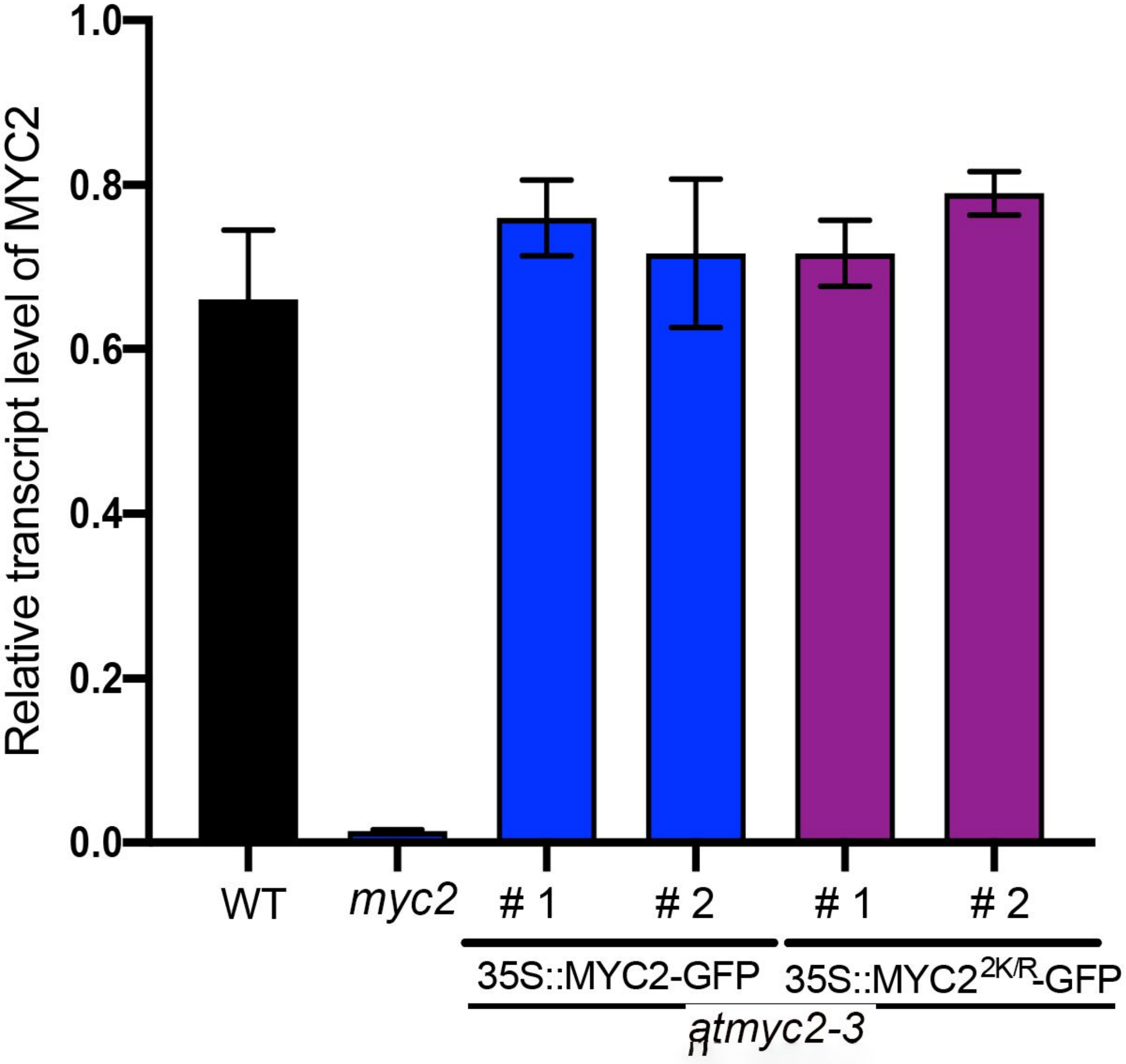

Supplemental Figure 5. Expression analysis of *MYC2* in transgenic lines of 35S: MYC2-GFP (*atmyc2-3*) (supports Figure 3). The expression levels of *MYC2* were ascertained in Col-0 and atmyc2-3 and two independent lines of 35S:MYC2-GFP (*atmyc2-3)* and 35S:MYC22K/R-GFP (*atmyc2-3*) by real time PCR analysis. The experiment was replicated three times and data shows mean ± SD (n=3). Error bars represent standard deviation and n stands for number of replicates.

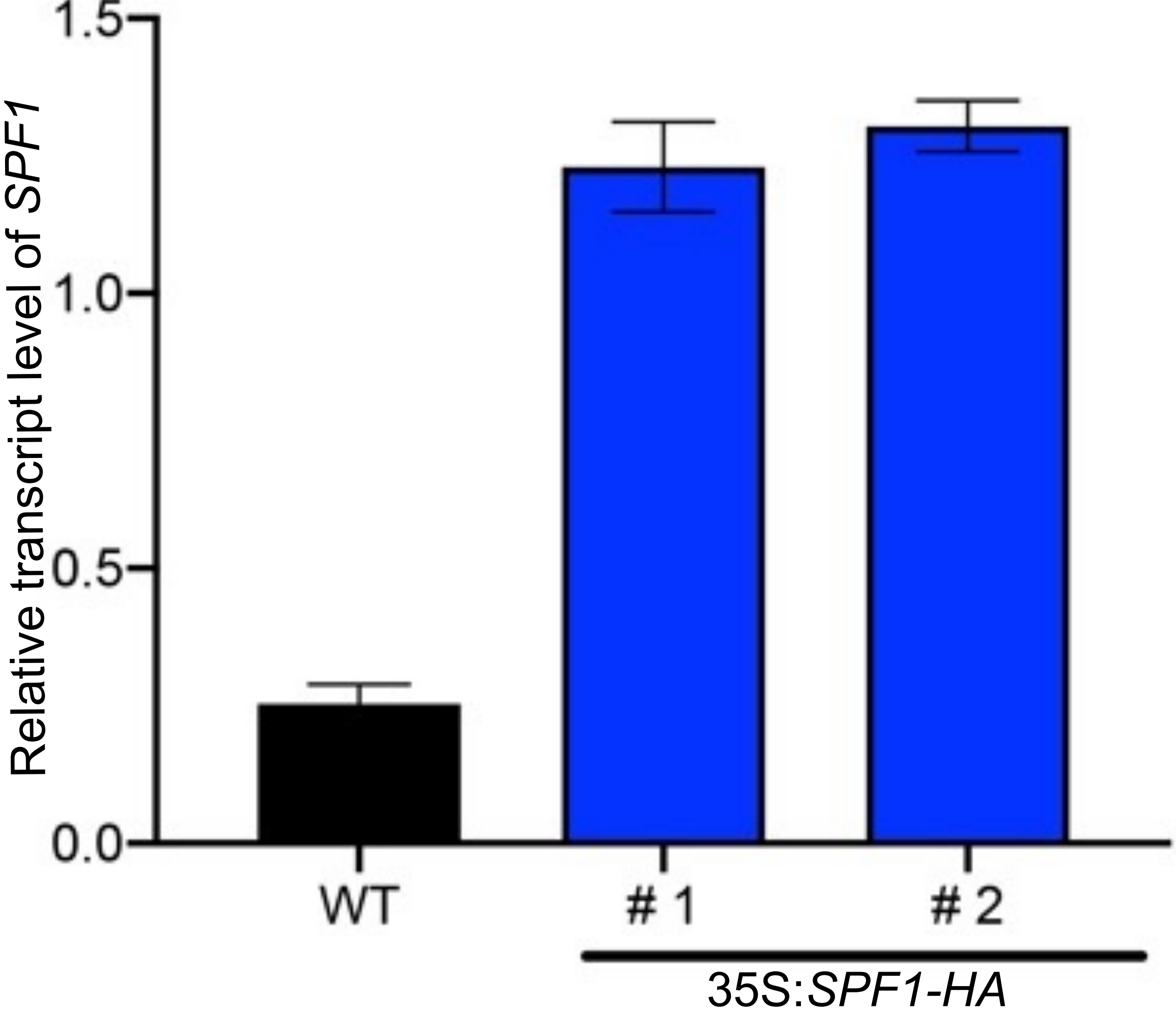

Supplemental Figure S6. Expression analysis of *SPF1* in 35S: *SPF1-HA* (*Col-0*) transgenic lines (supports Figure 4). The expression levels of *SPF1* were measured in Col-0 and two independent lines of 35S:*SPF1-HA* by RT-qPCR analysis. The experiment was replicated three times, and data show mean ± SD (n=3). Error bars represent standard deviation and n stands for number of replicates.

Supplemental Data. Srivastava et al. (2022). The conjugation of SUMO to the transcription factor MYC2 functions in blue light-mediated seedling development in Arabidopsis. Plant Cell.

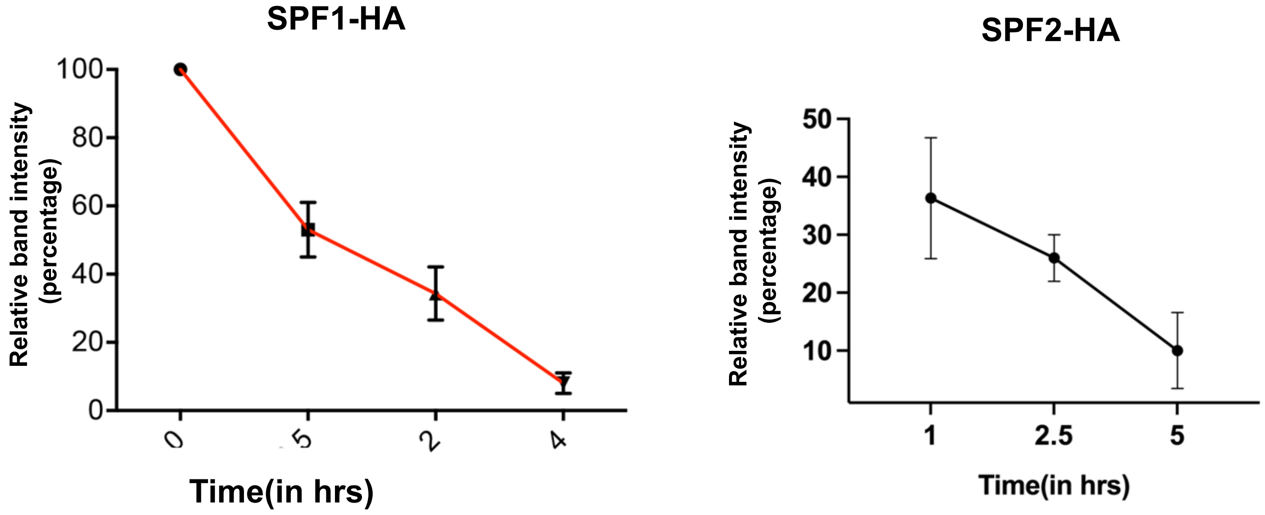

Supplemental Figure S7. Quantification of SPF1-HA and SPF2-HA in the immunoblots in Figure 4B and C using ImageJ software (supports Figure 4). The experiment was replicated three times, and data show mean ± SD (n=3). Error bars represent standard deviation and n represents number of replicates.

Supplemental Data. Srivastava et al. (2022). The conjugation of SUMO to the transcription factor MYC2 functions in blue light-mediated seedling development in Arabidopsis. Plant Cell.

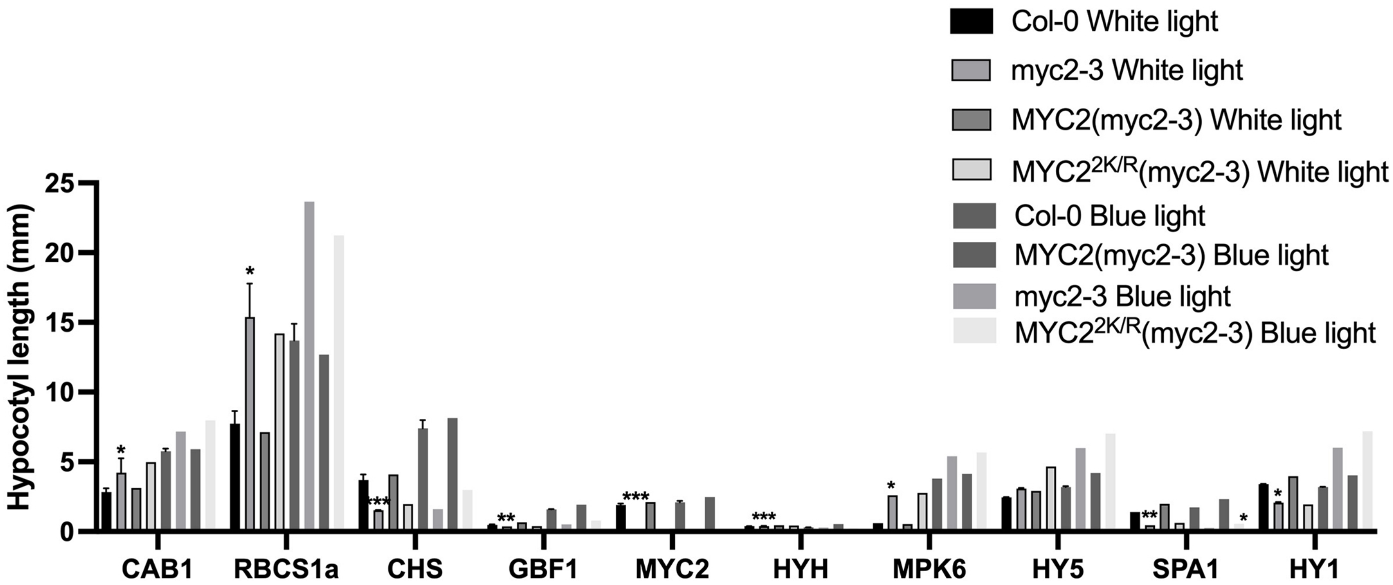

Supplemental Figure 8. Expression analysis of light responsive genes. Transcript level of CAB1, RBCS1a, CHS, GBF1, MYC2, HYH, MPK6, HY5, SPA1, HY1 in Col-0, atmyc2-3, 35S:MYC2-GFP, 35S:MYC2^{2KR}-GFP(myc2-3) under white light and blue light. RNA was extracted from six days old seedlings grown in constant white light or blue light.

Supplemental Data. Srivastava et al. (2022). The conjugation of SUMO to the transcription factor MYC2 functions in blue light-mediated seedling development in Arabidopsis. Plant Cell.

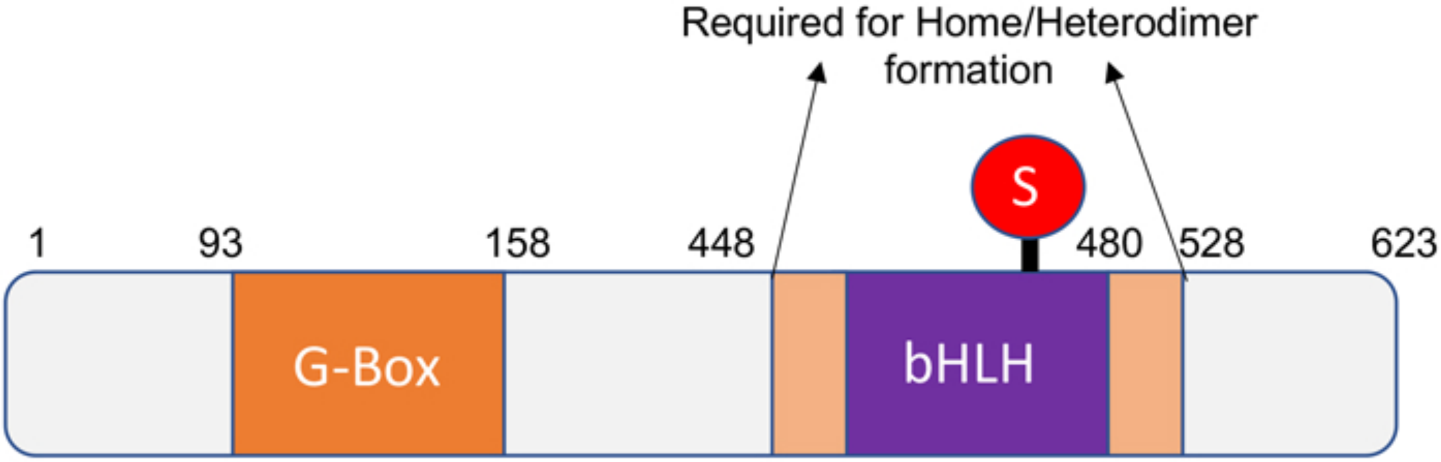

Supplemental Figure 9. Schematic depicting the organization of MYC2 structural domains (supports Figure 6). bHLH (basic Helix loop Helix domain) required for heterodimerization and binding to the G-box sequence in target promoters is indicated in purple and the SUMO binding sites is indicated by a red circle.

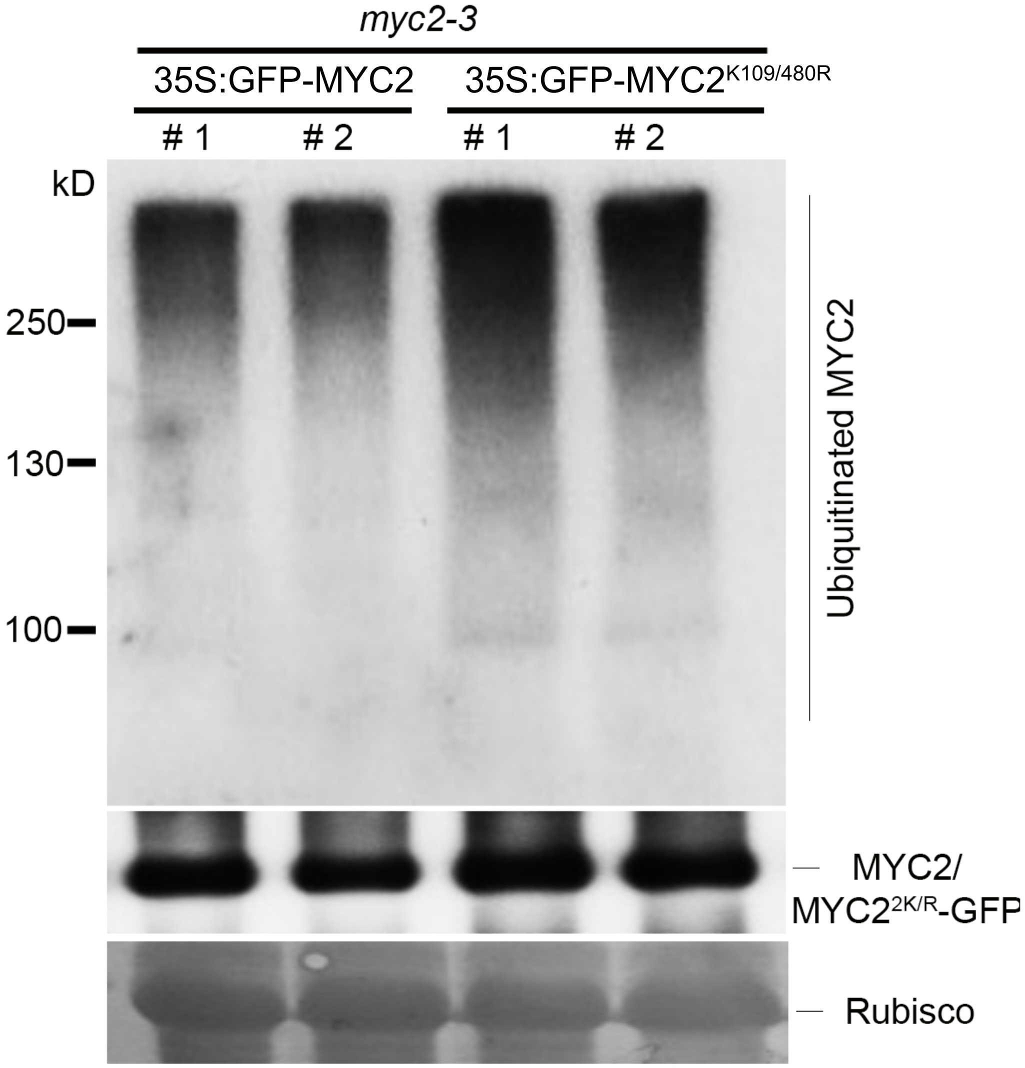

Supplemental Figure S10. Immunoblot detecting ubiquitination to illustrate the comparative ubiquitination of MYC2-GFP and MYC22K/R-GFP in stable Arabidopsis transgenic lines (supports Figure 7).
Total proteins were extracted for immunoprecipitation assays with αGFP antibody beads (IP: αGFP), followed by immunoblot analysis with α ubiquitin (IB: αUb) antibodies to detect ubiquitinated MYC2/MYC22K/R and αGFP (IB: αGFP) antibodies to detect MYC2/MYC22K/R GFP. GFP was used as a negative control. Ponceau staining for Rubisco was used to ascertain even protein loading in each lane.

Supplemental Table S1. List of DNA oligonucleotides used in the study.

Table of DNA primers employed to generate the DNA constructs and for RT-qPCR.

No.	Oligo name	Sequence	Purpose
1	MYC2 FL FP	CACCATGACTGATTACCGGCTACAACCAAC	Cloning
2	MYC2 FL RP	TTAACCGATTTTTGAAATCAAAC	Cloning
3	MYC3 FL RP	CGGATCCATGAACGGCACAACATCATCAATC	Cloning
4	MYC3 FL RP	GGAATTCATAGTTTTCTCCGACTTTCGTCATC	Cloning
5	PUB10 FL FP	CACCATGGCTGGTGGAGCTATCAC	Cloning
6	PUB10 FL RP	GAGTGAACCTAATTTTCGGGATGAT	Cloning
7	MYC2K109R FP	GGTTATTACAGAGGTGAAGAA	Site-directed mutagenesis
8	MYC2K109R RP	TTCTTCACCTCTGTAATAACC	Site-directed mutagenesis
9	MYC2K480R FP	AAAATGGATAGAGCTTCGTTA	Site-directed mutagenesis
10	MYC2K480R RP	TAACGAAGCTCTATCCATTTT	Site-directed mutagenesis
11	SPF1 FL FP	CACCATGAAGAAAAACTTTGAAGTATTC	Cloning
12	SPF1 FL RP	TTTCTCCATCTCCTCAGCTTCGCC	Cloning
13	SPF2 FL FP	CACCATGACTCTCCGGTCAGTTC	Cloning
14	SPF2 FL RP	AGTTTTTGGCTTGGCCATC	Cloning
15	CAB1 RT FP	GAGGAAGACTGTTGCCAAGC	qRT-PCR
16	CAB1 RT RP	CCCACCTGCTGTGGATAACTTC	qRT-PCR
17	RBCS-1A RT FP	ACCTTATCCGCAACAAGTGG	qRT-PCR
18	RBCS-1A RT RP	TGGGGTACTCCTTCTTGCAC	qRT-PCR
19	CHS RT FP	GGAAGAGAAGATGAGGGCGA	qRT-PCR
20	CHS RT RP	AACAAGACACCCCACTCCAA	qRT-PCR
21	GBF1 RT FP	TGGCAAGAAGTCAAAGGGGA	qRT-PCR
22	GBF1 RT RP	CCCTGTTCCTGTTGATTGGC	qRT-PCR
23	MYC2 RT FP	AGAACGACCCGTCTATGTGG	qRT-PCR
24	MYC2 RT RP	CTACCGTTTGCTGGCTTTCTT	qRT-PCR
25	HYH RT FP	GCAGCTGGATCAACATGTGT	qRT-PCR
28	HYH RT RP	TGCGCTGATACTCTGTTCCT	qRT-PCR
29	HY5 RT FP	CGGAGTTTGGAGGAGAAGCT	qRT-PCR
30	HY5 RT RP	CTCTTCAGCCGCTTGTTCTC	qRT-PCR
31	MPK6 RT FP	AGCCTCCGATCATGCCTATT	qRT-PCR
32	MPK6 RT RP	CATGTGACGAAGCAGCTTGA	qRT-PCR
33	SPA1 RT FP	ACTTGGCCTCGACTGACTAC	qRT-PCR
34	SPA1 RT RP	TCACCGAGCAGTCATCACTT	qRT-PCR
35	HY1 RT FP	TTGCTCATAGTGCTGGTGGA	qRT-PCR
36	HY1 RT RP	TAGTCCACTCCTCTGCAACC	qRT-PCR
37	LOX2 RT FP	CTTGCTCGTCCGGTAATTGG	qRT-PCR
38	LOX2 RT RP	TCTGAGGGAAGATGGAGGGA	qRT-PCR

40	VSP2 RT FP	GCGTACTGGTTGTGGTTAGG	qRT-PCR
41	VSP2 RT RP	CCAGCAGCTTCGAGATTGTC	qRT-PCR
