# Supplementary material for: The conjugation of SUMO to the transcription factor MYC2 functions in blue light-mediated seedling development in Arabidopsis
Source: Plant Cell. 2022 May 14;34(8):2892–906. doi: 10.1093/plcell/koac142 (PMC9338799; doi:10.1093/plcell/koac142)
Supplement: koac142_Supplementary_Data [file koac142_supplementary_data.zip › koac142-suppl_data/tpc.21.00535_SupplementalFile1.pdf]

Srivastava, et al. (2022). The conjugation of SUMO to the transcription factor MYC2 functions in blue light-mediated seedling development in Arabidopsis. Plant Cell.

Supplemental File S1. ANOVA tables

Supplemental Figure 1B

Blue light intensity (in µmolm^{-2} sec^{-1})	Mean hypocotyl length of Col-0(in mm)	Mean hypocotyl length of spf1spf2(in mm)	df	P value	Genotype
10	4.441	6.136	17.79	0.000004	Col-0 vs spf1/spf2
30	4.285	5.619	17.17	0.000029	Col-0 vs spf1/spf2
60	1.872	2.755	17.86	0.000154	Col-0 vs spf1/spf2
90	1.768	2.653	15.66	0.000059	Col-0 vs spf1/spf2
30	4.285	1.703	17.99	0.000049	Col-0 vs MYC$^{2K/R}$(myc2-3)

Supplemental Figure 1D

Far light intensity (in µmolm^{-2} sec^{-1})	Mean hypocotyl length of Col-0(in mm)	Mean hypocotyl length of spf1spf1(in mm)	df	P value	Genotype
0.5	2.112	2.895	27.32	<0.000001	Col-0 vs spf1/spf2
2	1.61	1.911	26.01	<0.000001	Col-0 vs spf1/spf2
5	1.504	2.404	27.01	0.000014	Col-0 vs spf1/spf2
10	2.303	3.83	28.01	<0.000001	Col-0 vs spf1/spf2
0.5	2.112	2.669	29.01	0.001618	Col-0 vs MYC2(spf1spf2)
2	1.161	1.881	30.01	0.000006	Col-0 vs MYC2(spf1spf2)
5	1.504	2.135	31.01	0.000002	Col-0 vs MYC2(spf1spf2)
10	2.303	2.871	32.01	0.0001	Col-0 vs MYC2(spf1spf2)

Supplemental 2A

Gene	Average expression value under white light	Average expression value under blue light	df	P value	Genotype
SPF1	0.01397	0.008393	4	0.0101	Col-0

Supplemental 2B
White light

Gene	Mean hypocotyl length of Col-0(in mm)	Mean hypocotyl length of spf1spf1(in mm)	df	P value	Genotype
CAB1	2.83	0.64	3.977	0.000515	Col-0 vs spf1spf2
RBCS1a	7.74	3.79	2.04	0.016111	Col-0 vs spf1spf2
CHS	3.7	1.7	3.816	0.00295	Col-0 vs spf1spf2
GBF1	0.48	0.01	2.018	0.001299	Col-0 vs spf1spf2
MYC2	1.91	6.5	2.16	0.002935	Col-0 vs spf1spf2
HYH	0.38	0.04	2.01	0.001123	Col-0 vs spf1spf2
MPK6	0.6	0.2	2.941	<0.000001	Col-0 vs spf1spf2
HY5	2.45	1.01	2.941	0.000015	Col-0 vs spf1spf2
SPA1	1.4	2.9	4	<0.000001	Col-0 vs spf1spf2
HY1	3.4	1.01	2.941	<0.000001	Col-0 vs spf1spf2

Blue light

Gene	Mean hypocotyl length of Col-0(in mm)	Mean hypocotyl length of spf1spf1(in mm)	df	P value	Genotype
CAB1	5.77	2.67	3.952	0.000033	Col-0 vs spf1spf2
RBCS1a	13.71	3.68	2.832	0.001273	Col-0 vs spf1spf2
CHS	7.4	2.7	2.439	0.002659	Col-0 vs spf1spf2
GBF1	1.6	0.04	2.04	0.000011	Col-0 vs spf1spf2
MYC2	2.1	6.6	2.111	0.004893	Col-0 vs spf1spf2
HYH	0.3	0.02	2.357	0.000151	Col-0 vs spf1spf2
MPK6	3.8	1.6	2.941	<0.000001	Col-0 vs spf1spf2
HY5	3.2	0.98	2.16	0.000098	Col-0 vs spf1spf2
SPA1	1.74	2.98	3.18	<0.000001	Col-0 vs spf1spf2
HY1	3.2	0.98	2.941	<0.000001	Col-0 vs spf1spf2
